# Calmodulin variant E140G associated with long QT syndrome impairs CaMKIIδ autophosphorylation and L-type calcium channel inactivation

**DOI:** 10.1016/j.jbc.2022.102777

**Published:** 2022-12-08

**Authors:** Ohm Prakash, Nitika Gupta, Amy Milburn, Liam McCormick, Vishvangi Deugi, Pauline Fisch, Jacob Wyles, N Lowri Thomas, Svetlana Antonyuk, Caroline Dart, Nordine Helassa

**Affiliations:** 1 Liverpool Centre for Cardiovascular Science, Department of Cardiovascular and Metabolic Medicine, Institute of Life Course and Medical Sciences, Faculty of Health and Life Sciences, University of Liverpool, Liverpool, United Kingdom; 2 Department of Molecular Physiology and Cell Signalling, Institute of Systems, Molecular and Integrative Biology, Faculty of Health and Life Sciences, University of Liverpool, Liverpool, United Kingdom; 3 School of Pharmacy & Pharmaceutical Sciences, Cardiff University, Cardiff, United Kingdom; 4 Molecular Biophysics Group, Institute of Systems, Molecular and Integrative Biology, Faculty of Health and Life Sciences, University of Liverpool, Liverpool, United Kingdom

**Keywords:** calmodulin, Ca^2+^/calmodulin-dependent protein kinase II, CaMKIIδ, cardiac arrhythmia, long QT syndrome, LQTS, L-type voltage-gated Ca^2+^ channel, Ca_v_1.2, CaM, calmodulin, CDF, Ca^2+^-dependent facilitation, CDI, Ca^2+^-dependent inactivation, ITC, isothermal titration calorimetry, LQTS, Long QT syndrome, SR, sarcoplasmic reticulum

## Abstract

Long QT syndrome (LQTS) is a human inherited heart condition that can cause life-threatening arrhythmia including sudden cardiac death. Mutations in the ubiquitous Ca^2+^-sensing protein calmodulin (CaM) are associated with LQTS, but the molecular mechanism by which these mutations lead to irregular heartbeats is not fully understood. Here, we use a multidisciplinary approach including protein biophysics, structural biology, confocal imaging, and patch-clamp electrophysiology to determine the effect of the disease-associated CaM mutation E140G on CaM structure and function. We present novel data showing that mutant-regulated CaMKIIδ kinase activity is impaired with a significant reduction in enzyme autophosphorylation rate. We report the first high-resolution crystal structure of a LQTS-associated CaM variant in complex with the CaMKIIδ peptide, which shows significant structural differences, compared to the WT complex. Furthermore, we demonstrate that the E140G mutation significantly disrupted Ca_v_1.2 Ca^2+^/CaM-dependent inactivation, while cardiac ryanodine receptor (RyR2) activity remained unaffected. In addition, we show that the LQTS-associated mutation alters CaM’s Ca^2+^-binding characteristics, secondary structure content, and interaction with key partners involved in excitation-contraction coupling (CaMKIIδ, Ca_v_1.2, RyR2). In conclusion, LQTS-associated CaM mutation E140G severely impacts the structure-function relationship of CaM and its regulation of CaMKIIδ and Ca_v_1.2. This provides a crucial insight into the molecular factors contributing to CaM-mediated arrhythmias with a central role for CaMKIIδ.

Long QT Syndrome (LQTS) is a life-threatening inherited cardiac arrhythmia with a predicted prevalence of approximately 1 in 2500 (1). It is characterized by prolongation of the QT interval on an ECG (2), caused by gain-of-function or loss-of-function mutations in cardiac ion channels that lead to prolongation of cellular repolarization (3). More than 90% of genetically confirmed cases of LQTS can be linked to mutations in potassium (K^+^) or sodium (Na^+^) channels (4, 5, 6). However, mutations in the highly conserved calcium (Ca^2+^)-sensing protein, calmodulin (CaM), have recently been implicated in LQTS (5, 7, 8, 9, 10, 11, 12, 13, 14, 15, 16, 17, 18, 19). CaM variant–mediated LQTS present different disease severities and this phenotypic presentation may be regulated by distinct underlying mechanisms. It is important to understand the mechanistic differences between CaM variants and their associated phenotype for targeted therapies.

CaM is a Ca^2+^ sensing protein able to bind up to four Ca^2+^ ions through its Ca^2+^-binding EF-hand motifs: two located in its N-domain (EF-1 and EF-2) and two in its C-domain (EF-3 and EF-4). The associated structural transition to an open conformation upon Ca^2+^ binding mediates interaction with CaM’s targets and signal transduction (20, 21). In cardiomyocytes, CaM modulates the activity of several ion channels such as the L-type voltage-gated Ca^2+^ channel (Ca_v_1.2), voltage-gated Na^+^ channel (Na_v_1.5), voltage-gated K^+^ channel (K_v_7.1), and ryanodine receptor (RyR2) (22, 23, 24, 25, 26, 27, 28, 29, 30, 31, 32, 33, 34, 35, 36, 37, 38, 39, 40, 41, 42, 43, 44, 45). Modulation is achieved either *via* direct binding or through the regulatory multifunctional Ca^2+^/CaM-dependent kinase (CaMKII) with the γ (CaMKIIγ) and δ (CaMKIIδ) isoforms present in heart (46, 47, 48, 49).

Opening of Na_v_1.5 in cardiomyocytes results in cell membrane depolarization and activation of Ca_v_1.2. Inward flux of Ca^2+^ into the cytoplasm *via* Ca_v_1.2 triggers the opening of RyR2 embedded in the sarcoplasmic reticulum. This causes release of internal Ca^2+^ stores through a process called Ca^2+^-induced Ca^2+^ release (50). Ca^2+^-induced Ca^2+^ release increases the cytosolic free Ca^2+^ concentration ([Ca^2+^]cyt) throughout the cardiomyocyte and binding of Ca^2+^ to myofilaments results in contraction. At high [Ca^2+^]_cyt_, Ca^2+^/CaM binding to RyR2 causes inhibition of RyR2 Ca^2+^ release (51). High [Ca^2+^]_cyt_ also inactivates the Ca_v_1.2 channel by Ca^2+^/CaM-dependent inactivation (CDI) and terminates Ca^2+^ entry to avoid Ca^2+^ overload and arrhythmias (22). Ca^2+^ triggers a second process called Ca^2+^-dependent facilitation (CDF) to offset partly reduced Ca^2+^ channel availability at high heart rates (52). Both types of regulation involve direct binding of Ca^2+^/CaM to the Ca_v_1.2 and RyR2 channels. Ca^2+^ reuptake into the SR and transport across the cell membrane into the extracellular space returns the cardiomyocyte to resting intracellular Ca^2+^ conditions. This allows Ca^2+^ dissociation from contractile proteins and cardiomyocyte relaxation.

CaM additionally modulates the activity of ion channels through CaMKII (46, 53). Under basal conditions, CaMKII remains in an inactive conformation due to the intramolecular interaction between its regulatory domain and catalytic domain (54). This inhibitory interaction prevents the substrate and ATP from binding to the catalytic domain of CaMKII. At high [Ca^2+^], Ca^2+^/CaM binding to CaMKII releases the regulatory domain from the catalytic domain. This allosteric rearrangement allows ATP and other substrates to gain access to the catalytic domain, thus allowing CaMKII to autophosphorylate itself at Thr287 and to phosphorylate downstream targets. Autophosphorylation, in addition to increasing the affinity of CaM–CaMKII interaction by 1000-fold *via* CaM trapping (55), maintains the catalytic activity of CaMKII by hindering the inhibitory reassociation between the regulatory domain and catalytic domains. This can occur in the absence of CaM binding. The downstream targets of CaMKII include Ca_v_1.2 and RyR2 channels. Phosphorylation by CaMKII increases the open probability of Ca_v_1.2 channels, thereby playing a role in CDF (56, 57). CaMKII can also phosphorylate RyR2 at S2814 and regulate CaM-mediated inhibition of the channel (46).

Based on the extensive roles of CaM in the direct and indirect regulation of cardiac excitation contraction coupling, we hypothesize that CaM mutations can disrupt its regulatory functions resulting in cardiac arrhythmia. Here, we report the results of a comprehensive functional, biophysical, and structural analysis performed to determine the effect of LQTS-associated CaM mutation E140G on CaMKIIδ, Ca_v_1.2, and RyR2. We obtained the X-ray structure of CaM-E140G in complex with CaMKIIδ peptide and show for the first time that the mutation induces conformational changes which affect CaM binding to CaMKIIδ and CaMKIIδ (auto)phosphorylation activity. We present novel electrophysiology and Ca^2+^ imaging data supporting that the E140G mutation significantly impairs Ca_v_1.2 CDI, while RyR2 activity is unchanged. The data obtained help decipher the molecular mechanism of CaM-associated LQTS and highlights CaMKIIδ as a key player.

## Results

### CaM-E140G variant impairs CaMKIIδ kinase activity and autophosphorylation

**Figure 1:**
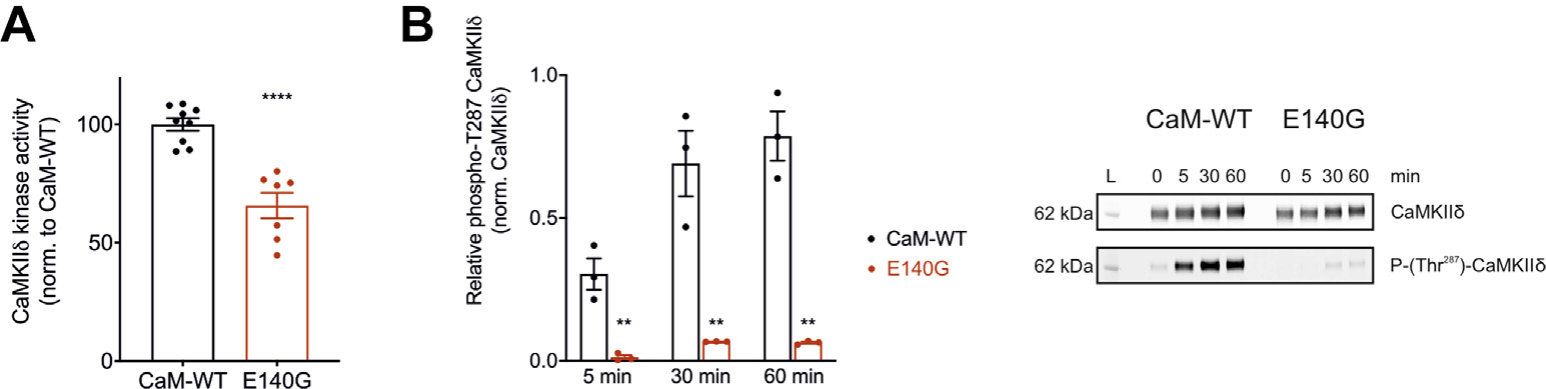
**Arrhythmic variant CaM-E140G decreases kinase activity and autophosphorylation of CaMKIIδ.***A*, quantification of phosphorylation activity of CaMKIIδ using Amplite universal fluorimetric kinase assay kit. (*B*-*left panel*), measurement of the relative levels of CaMKIIδ Thr287 autophosphorylation. GST-CaMKIIδ was incubated with CaM variants and ATP for 0 min, 5 min, 15 min, 30 min, and 60 min at room temperature. CaM-WT or CaM-E140G recombinant proteins were used as CaMKIIδ activators. The reaction was terminated using SDS-containing solution, and samples were analyzed by Western blotting and densitometry analysis. (*B*-*right panel*), representative blots for CaM-WT and CaM-E140G samples. Phosphorylated proteins (phospho-Thr287 antibody) were normalized to total CaMKIIδ protein (GST antibody). Experiments were performed at least in triplicates. Data are expressed as mean ± s.e.m. Differences between groups were determined using a two-tailed unpaired Student *t* test. *p*-values are represented by stars with ∗∗*p* < 0.01 and ∗∗∗∗*p* < 0.0001. CaM, calmodulin.

In order to evaluate the functional impact of the Ca^2+^/CaM-E140G variant on CaMKIIδ kinase activity, we measured the amount of ADP produced using an end point fluorimetric assay. Using syntide2 as the enzyme substrate, commercial human CaMKIIδ (Abcam) as the enzyme, and CaM variants as activators, we find that phosphorylation levels decreased by ∼35% (Fig. 1*A*) for E140G when compared to CaM-WT. Further, the rate of CaMKIIδ autophosphorylation (Thr287) was determined using Western blotting and densitometry analysis (Figs. 1*B* and S1). After a 5 min of reaction (incubation of CaMKIIδ with Ca^2+^/CaM variants and ATP, at room temperature), the relative CaMKIIδ autophosphorylation level at Thr287 was significantly decreased from 0.30 ± 0.05 (WT) to 0.01 ± 0.008 (E140G). We determined that even after a 60 min reaction, E140G showed ∼13-fold lower CaMKIIδ autophosphorylation levels than CaM-WT. These data suggest that the decrease in kinase activity could be attributed to impaired CaMKIIδ autophosphorylation in the presence of CaM-E140G variant.

### LQTS-associated E140G mutation disrupts interaction with CaMKIIδ

**Figure 2:**
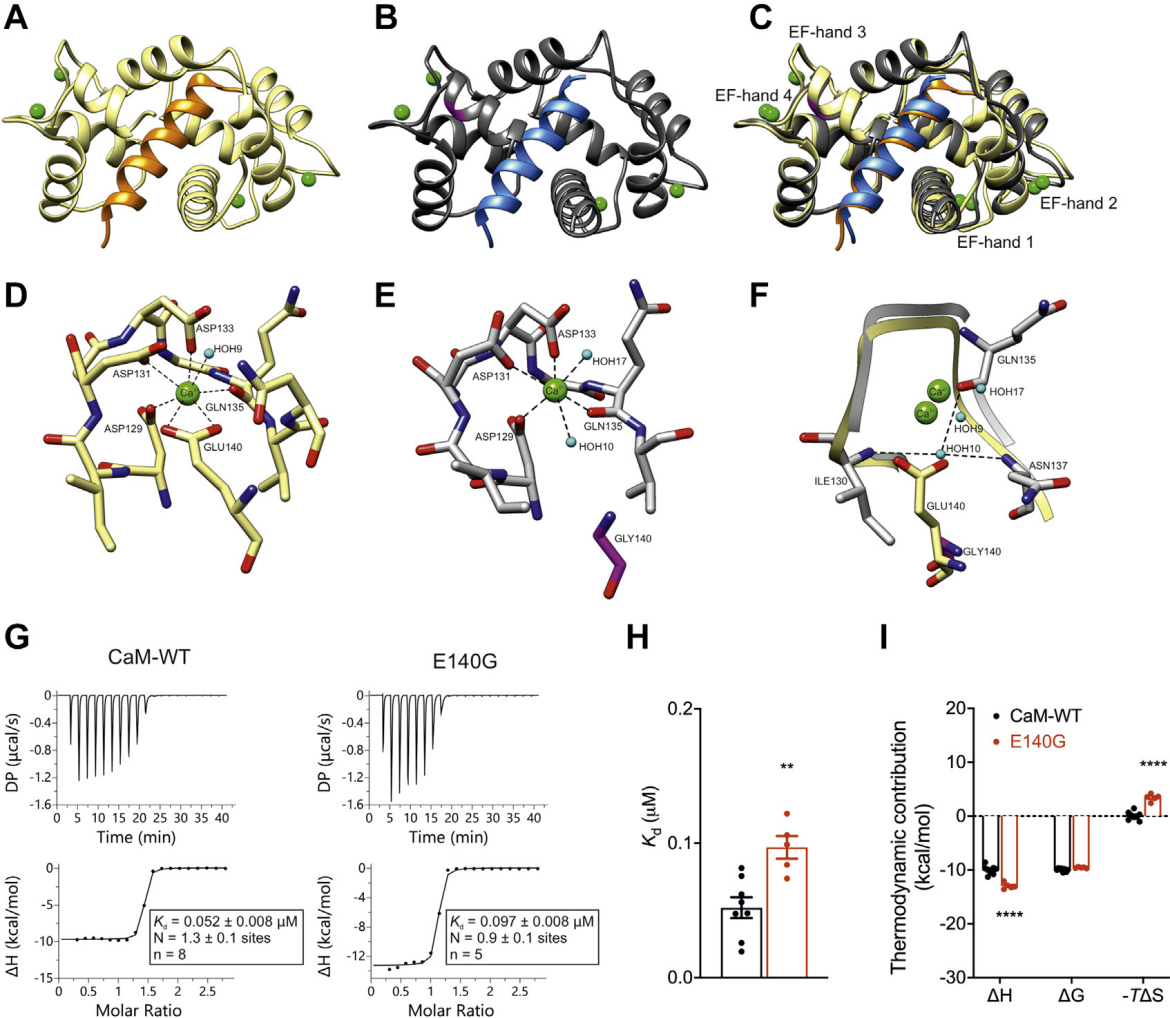
**E140G mutation disrupts interaction of CaM with CaMKIIδ.***A*, cartoon representation of the crystal structure of CaM-WT (*yellow*) in complex with CaMKIIδ_294-315_ (*orange*). *B*, cartoon representation of the crystal structure of CaM-E140G (*gray*) in complex with CaMKIIδ_294-315_ (*blue*). *C*, structural superimposition of CaM-WT and CaM-E140G in complex with CaMKIIδ_294-315_. *D* and *E*, H-bond interactions between Ca^2+^ and CaM residues at the EF hand 4 in the (*D*) Ca^2+^/CaM-WT–CaMKIIδ_294-315_ and (*E*) Ca^2+^/CaM-E140G–CaMKIIδ_294-315_ complex structure. *F*, superimposition of EF-hand 4 region of Ca^2+^/CaM-WT–CaMKIIδ_294-315_ and Ca^2+^/CaM-E140G–CaMKIIδ_294-315_ complex structures. The Glu140:OE2 atom is replaced by a water molecule in the Ca^2+^/CaM-E140G–CaMKIIδ_294-315_ complex mutant structure to coordinate the Ca^2+^. Ca^2+^ is shown in *green* sphere and water molecule in *cyan* sphere. H-bond is represented in *black dashed lines*. G140 is shown in *magenta*. Images were created with UCSF Chimera (112). *G*, representative ITC titration curves (*upper panels*) and binding isotherms (lower panels) for Ca^2+^–CaM interaction with CaMKIIδ_294-315_. *H*, affinity of the binding of Ca^2+^/CaM with CaMKIIδ_294-315_ obtained by fitting to a one-site–binding model. Differences between groups were determined using two-tailed unpaired Student *t* test. *I*, thermodynamic profile of binding between Ca^2+^/CaM and CaMKIIδ_294-315_. Data were processed using the MicroCal PEAQ-ITC software. *K*_d_, binding affinity; N, stoichiometry; n, number of experimental replicates. The sum of the change in enthalpy (ΔH) and the change in entropy (ΔS) multiplied by the absolute temperature (T) gives the change in free energy (ΔG). ITC experiments were performed in the presence of 5 mM CaCl_2_ at 25 °C. Data are mean±s.e.m. Differences between groups were determined using a two-way ANOVA with Sidak’s multiple comparisons test. *p*-values are represented by stars with ∗∗*p* < 0.01 and ∗∗∗∗*p* < 0.0001. The ANOVA parameters are shown in Table S3. CaM, calmodulin; DP, differential power; ITC, isothermal titration calorimetry.

Ca^2+^/CaM and the E140G mutant were cocrystallized with CaMKIIδ_294-315_ to gain molecular insight into the interaction. The crystal structures were solved to a high resolution of 2.65 Å for Ca^2+^/CaM-WT–CaMKIIδ_294-315_ complex (PDB 7ZRP) and 1.68 Å for Ca^2+^/CaM-E140G–CaMKIIδ_294-315_ complex (PDB 7ZRQ) (Fig. 2, *A*–*C*). Crystallographic data and refinement statistics are presented in Table 1. Both complexes gave clear electron density for four Ca^2+^ ions at the N- and C-domains of CaM. The structural superimposition Ca^2+^/CaM-WT-CaMKIIδ_294-315_ with Ca^2+^/CaM-E140G-CaMKIIδ_294-315_ showed rather low RMSD of 0.949 Å with difference mainly at the N-terminal region of CaM. Almost one-half turn of the helix in the region Phe65 to Lys77 is missing from the E140G complex.

**Table 1:** Data collection and refinement statistics

X-ray diffraction data and refinement	Ca^2+^/CaM-WT–CaMKIIδ_294-315_	Ca^2+^/CaM-E140G–CaMKIIδ_294-315_
Data collection Wavelength (Å)	0.97951	0.97951
Beamline	I04	I04
Detector	Dectris Eiger2 XE	Dectris Eiger2 XE
Space group	P2_1_2_1_2_1_	P2_1_2_1_2_1_
Unit-cell dimensions (*a,b,c*) (Å)	64.25, 72.50, 77.95	41.38, 54.52, 57.5
Resolution (Å)	2.65–53.19 (2.78–2.65)	39.56–1.68 (1.71–1.68)
*Rmerge* %	16.6 (214.4)	5.3 (242.1)
*Rpim %*	0.102 (1.249)	0.030 (1.452)
*I*/*σ* (last shell)	5.9 (0.7)	13.5 (0.6)
Completeness (%)	100.0 (100.0)	99.1 (87.8)
Redundancy	5.8 (6.1)	6.3 (4.6)
Half-set correlation CC_1/2_	0.996 (0.576)	1.000 (0.311)
No. of unique reflections	11133	15287
*R*_work_*/R*_free_	20.7/27.2	20.7/24.7
No. of atoms		
Protein	2636	1302
Ions	17	4
Water	36	40
B factor (Å^2^)		
Protein	65.4	31.2
Ions	72.1	23.6
Waters	52.8	48.1
R.M.S deviations		
Bond length (Å)	0.008	0.008
Bond angles (°)	1.49	1.50
PDB code	7ZRP	7ZRQ

Values in brackets are for the last resolution shell.

However, subtle changes were also observed in the C-domain, more specifically in EF-hand 4 when comparing the Ca^2+^/CaM-WT–CaMKIIδ_294-315_ and Ca^2+^/CaM-E140G–CaMKIIδ_294-315_ peptide complex structure (Fig. 2, *D*–*F*). Glutamate at position 140 (E140) is one of the Ca^2+^ coordinating residues in EF-hand 4. Interestingly, in the LQTS variant structure (G140), this interaction is lost and replaced by a water molecule, HOH10, coordinated by the residues I130, N137, and Q135. The H-bonds and salt bridge interactions between the CaMKIIδ peptide and CaM were predicted using the QtPISA server (58). The E140G mutation induced subtle differences in both H-bonds and salt bridge interactions (Tables S1 and S2). Ca^2+^/CaM-E140G–CaMKIIδ_294-315_ showed two extra unique H-bonds between Ala147[O]:Lys8[NZ] and Glu87[OE1]:Thr18[OG1] compared to Ca^2+^/CaM-WT-CaMKIIδ_294-315_. The Met144[O]:Arg4[NH2] H-bond in WT complex is replaced by Ala147[O]:Arg4[NE] H-bond in the mutant structure. The Lys75[NZ]:Met15[O] H-bond in the WT complex is absent in the mutant complex.

Using isothermal titration calorimetry (ITC), we investigated the binding of CaM variants to CaMKIIδ-binding domain. ITC provides the dissociation constant (*K*_d_) and the stoichiometry of binding (N) of interactions. In addition, from the thermodynamic parameters, the nature of the forces that drive the binding reaction can be determined (enthalpy change, ΔH and the entropic term ΔS). Using ITC, we showed that Ca^2+^-CaM can interact with CaMKIIδ_294-315_, with a stoichiometry of 1 (Fig. 2*G*). The dissociation constant of Ca^2+^/CaM for CaMKIIδ_294-315_ (0.052 ± 0.008 μM, n = 8) was significantly increased to 0.097 ± 0.008 μM for the E140G variant (n = 5), indicating a ∼2-fold reduction in binding affinity (Fig. 2*H*). The binding reaction was exothermic and enthalpy driven, with a significant increase of ΔH for CaM-E140G when compared to WT (Fig. 2*I*).

### CaM-E140G variant does not affect the voltage-dependence of Ca_v_1.2 activation and inactivation

**Figure 3:**
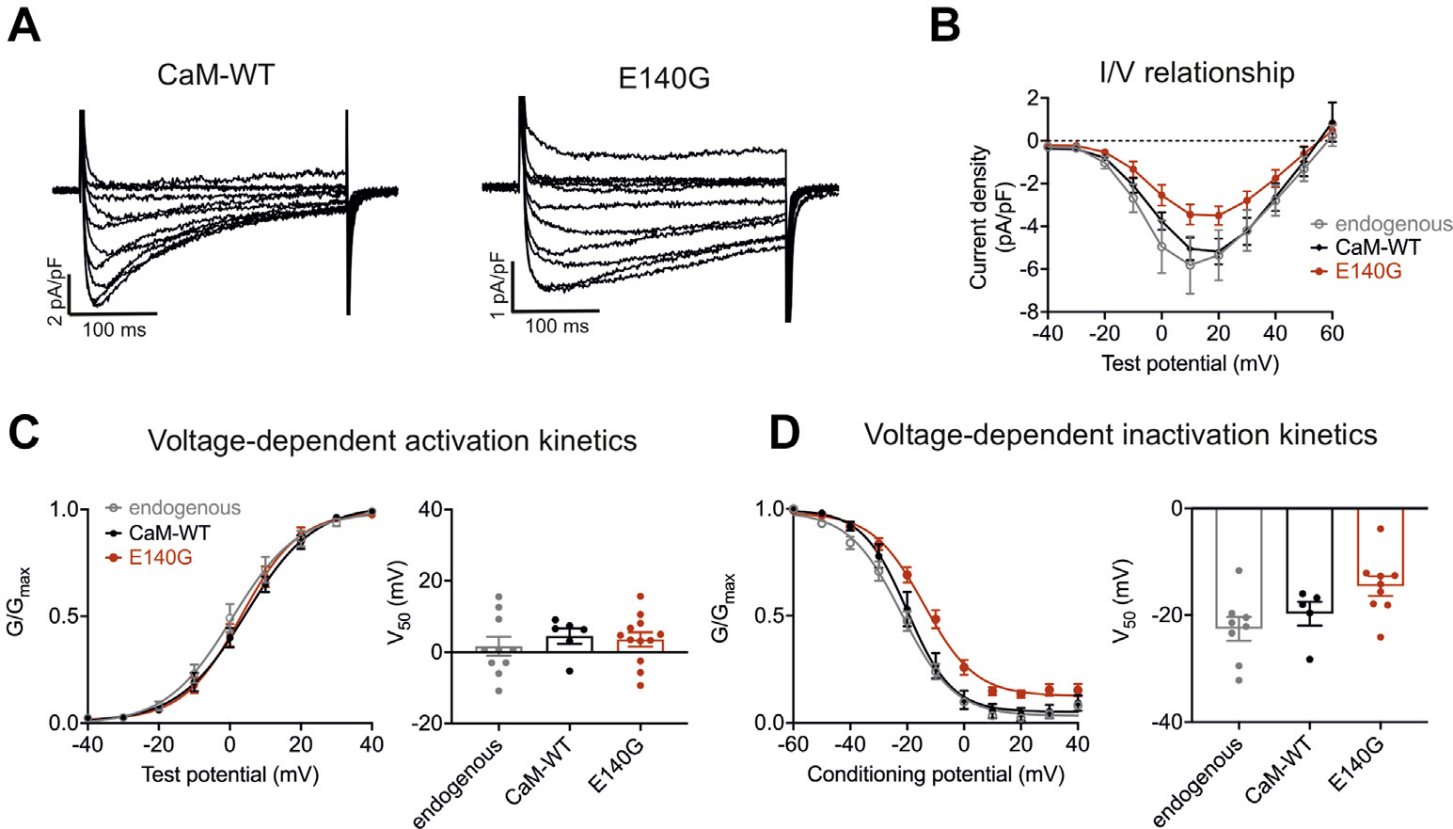
**LQTS-associated CaM variant E140G does not affect Ca**_**v**_**1.2 voltage-dependent activation characteristics.***A*, representative traces from HEK-Ca_v_1.2 cells–transfected CaM-WT or CaM-E140G and (*B*) I/V relationships of untransfected cells (endogenous) or cells transfected with CaM-WT or E140G. Differences between groups were determined using a two-way ANOVA with Tukey’s multiple comparisons tests. The ANOVA parameters are shown in Table S4. *C*, voltage-dependent activation characteristics of untransfected cells or cells transfected with CaM-WT or E140G. *D*, voltage-dependent inactivation characteristics of untransfected cells or cells transfected with CaM-WT or E140G. (*C* and *D*-*left panel*) mean (±s.e.m.) channel conductances, G, normalized to peak conductance, G_max_, to give mean activation/activation curves. (*C*, and *D*-*right panel*) mean (±s.e.m.) half maximal activation/inactivation voltages, V_50_, calculated from individual curves fitted using the Boltzmann equation. Experiments were performed in 0.5 mM EGTA (internal solution) and 2 mM CaCl_2_ (external solution). Differences between groups were determined using a one-way ANOVA with Dunnett’s multiple comparisons tests. The ANOVA parameters are shown in Tables S5 and S6. CaM, calmodulin; LQTS, Long QT syndrome.

In order to investigate the effect of the LQTS-associated variant CaM E140G on the voltage-dependent characteristics of Ca_v_1.2 whole-cell currents (I_Cav1.2_), patch-clamp electrophysiology was performed on HEK293-Ca_v_1.2 cells transiently transfected to overexpress CaM-WT or E140G (Fig. 3*A*). In these experiments, CaM variants and the fluorescent marker (EGFP) were coexpressed under the control of the same promoter, as two distinct proteins and not as fusion proteins. We observed a bell-shaped current-voltage relationship, which is characteristic of I_Cav1.2_ (Fig. 3*B*). The maximum peak current densities (measured at + 20 mV) were as follows: −5.34 ± 1.18 pA/pF (endogenous, n = 10); −5.17 ± 0.60 pA/pF (CaM-WT, n = 6), and -3.50 ± 0.43 pA/pF (E140G, n = 12). Using a two-way ANOVA with Tukey’s multiple comparisons test, we showed that the peak current densities at every voltage were not significantly affected by the overexpression of CaM-WT or the mutation. For both CaM-WT and E140G, activation curves showed a conductance increase from approximately −20 mV, with a maximum conductance achieved at the +40 mV test potential. V_50_ of activation, the voltage at which half-maximal conductance is reached, remained unchanged for E140G (3.59 ± 1.98 mV, n = 12) when compared to CaM-WT (4.52 ± 2.11 mV, n = 6) (Fig. 3*C*). V_50_ of voltage-dependent inactivation did not show any significant difference between CaM-WT (-19.71 ± 2.22 mV, n = 6) and E140G (-14.55 ± 1.84 mV, n = 12) (Fig. 3*D*). Together, this indicates that LQTS-associated CaM variant E140G does not affect the voltage-dependence of Ca_v_1.2 activation and inactivation.

### LQTS-associated variant CaM-E140G reduces CDI of Ca_v_1.2

**Figure 4:**
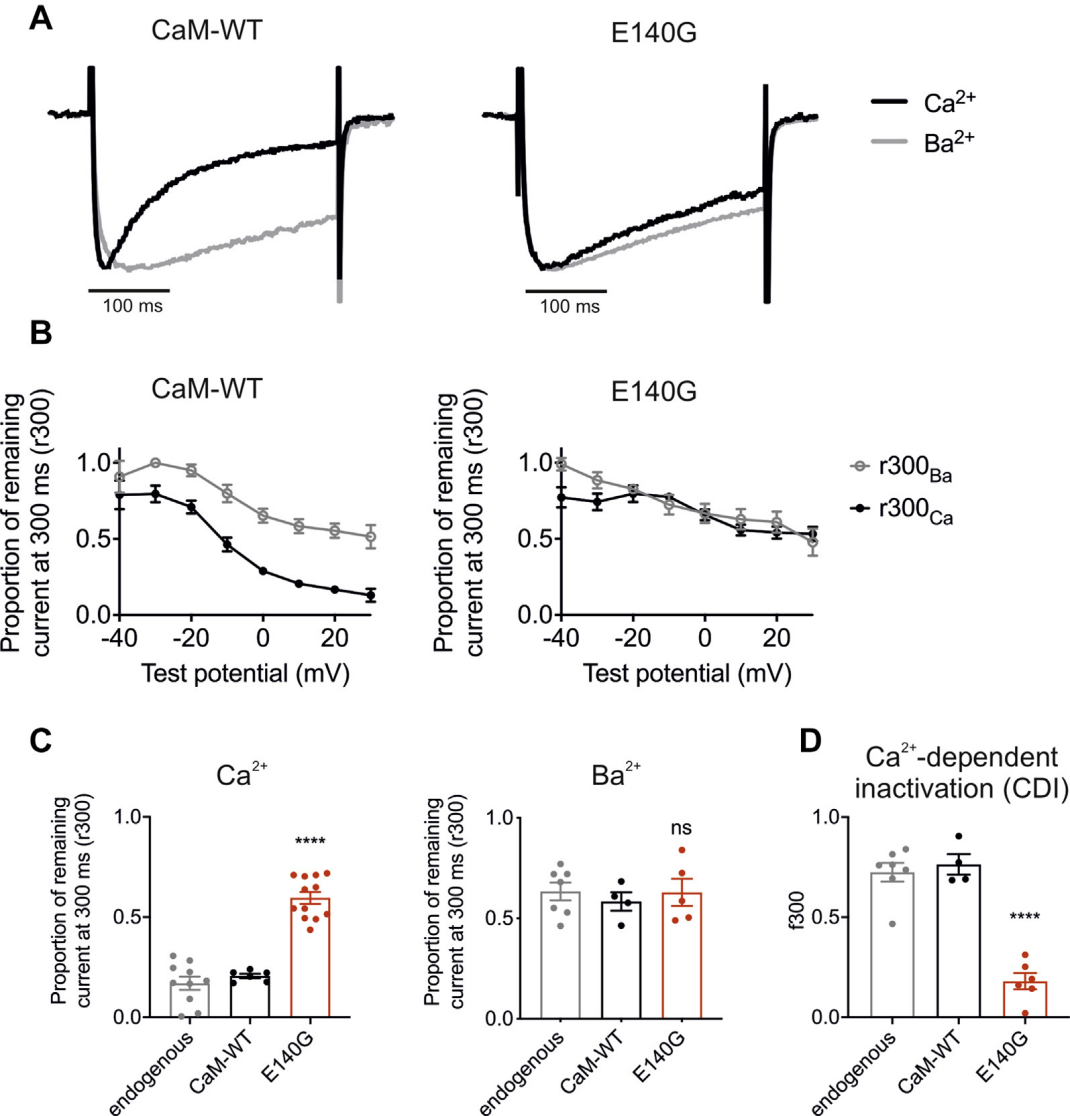
**LQTS-associated CaM variant E140G impairs Ca**_**v**_**1.2 Ca**^**2+**^**-dependent inactivation.***A*, representative Ca^2+^ (*black*) and Ba^2+^ (*gray*) current traces from HEK-Ca_v_1.2 cells either transfected with CaM-WT or E140G, in response to a 300 ms pulse to +10 mV, normalized to their respective peak currents. *B*, mean (±s.e.m.) fractional residual Ca^2+^ and Ba^2+^ current at the end of the 300 ms pulse (r300), at test potentials ranging from −40 to +20 mV. *C* and *D*, Ca^2+^-dependent and Ca^2+^-independent inactivation characteristics. (*C*-*left panel*), mean (±s.e.m.) residual Ca^2+^ current at the end of a 300 ms pulse (r300_Ca_), at +10 mV. (*C*-*right panel*), mean (±s.e.m.) residual Ba^2+^ current at the end of a 300 ms pulse (r300_Ba_), at +10 mV. *d*, mean (±s.e.m.) proportion of inactivation due to CDI (f300), at +10 mV. Experiments were performed in 0.5 mM EGTA (internal solution) and 2 mM of either CaCl_2_ or BaCl_2_ (external solution). Differences between groups were determined using using a one-way ANOVA with Dunnett’s multiple comparisons test. *p*-values are represented by stars with ∗∗∗∗*p* < 0.0001. The ANOVA parameters are shown in Tables S7 and S8. CaM, calmodulin; CDI, Ca^2+^-dependent inactivation; LQTS, Long QT syndrome.

Whole-cell recordings showed that CDI during the 300 ms depolarizing test pulse was impaired in cells overexpressing CaM E140G (Fig. 4*A*). In order to discriminate between Ca^2+^-dependent and Ca^2+^-independent inactivation, residual currents at the end of the test pulse were measured in the presence of either extracellular Ca^2+^ (r300_Ca_) or under conditions where all the extracellular Ca^2+^ was replaced by Ba^2+^ (r300_Ba_). Here, Ba^2+^ is able to enter the cell through the activated channel but is unable to induce CDI. Comparison of r300_Ca_ and r300_Ba_ across a range of different test potentials revealed a gradual decrease in r300_Ba_ with increasing depolarization for both CaM-WT and E140G (Fig. 4*B*). Mean r300_Ba_ at +10 mV for E140G remained unchanged, whereas mean r300_Ca_ significantly increased for E140G from 0.21 ± 0.01 (n = 6) to 0.60 ± 0.03 (n = 12), when compared to CaM-WT (Fig. 4*C*). The difference between r300_Ca_ and r300_Ba_, which indicates the proportion of total inactivation due to CDI, is denoted as f300 (Fig. 4*D*). A significant reduction in f300 for E140G was observed, from 0.76 ± 0.05 for WT (n = 4) to 0.18 ± 0.04 in E140G (n = 6). These data demonstrate a dramatic impairment in CDI in E140G, while Ca^2+^-independent inactivation is not affected (Fig. 3*D*).

### LQTS-associated E140G mutation disrupts interaction with Ca_v_1.2-binding domains

**Figure 5:**
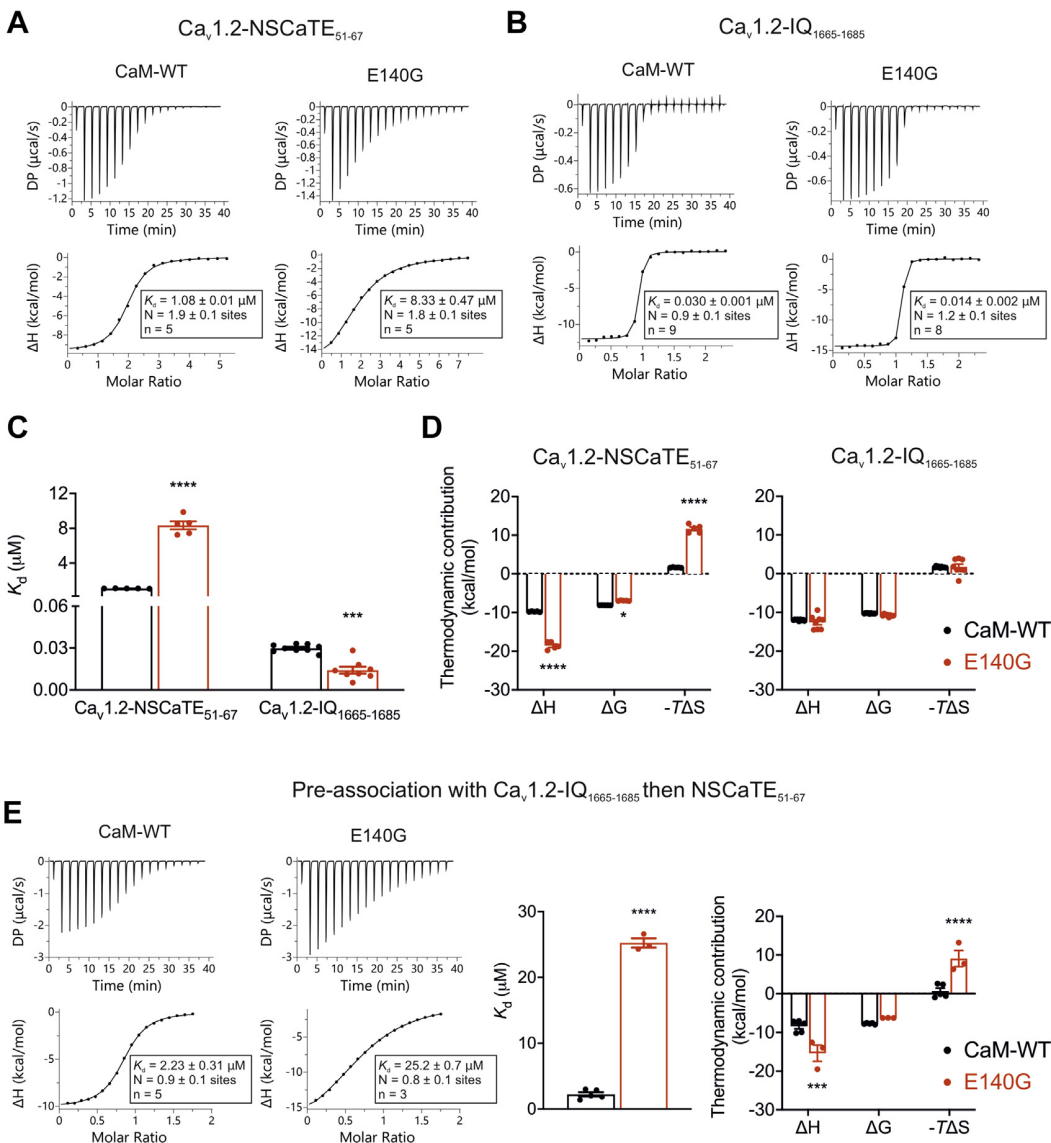
**LQTS-associated E140G mutation alters interaction of CaM with Ca**_**v**_**1.2-binding domains.***A* and *B*, representative ITC titration curves (*upper panels*) and binding isotherms (*lower panels*) for CaM interaction with (*A*) Ca_v_1.2-NSCaTE_51-67_ and (*B*) Ca_v_1.2-IQ_1665-1685_ peptides in the presence of Ca^2+^. *C*, affinity of the binding of Ca^2+^/CaM proteins with Ca_v_1.2 peptides. *D*, thermodynamic profile of binding between Ca^2+^/CaM proteins and Ca_v_1.2-NSCaTE_51-67_ (*left panel*), Ca_v_1.2-IQ_1665-1685_ (*right panel*). (*E*-*left panel*), representative ITC titration curves (*upper panels*) and binding isotherms (*lower panels*) for pre-associated CaM–Ca_v_1.2-IQ_1665-1685_ titrated with Ca_v_1.2-NSCaTE_51-67_ in the presence of Ca^2+^. (*E*-*middle panel*), affinity of binding and (*E*-*right panel*) thermodynamic profile for Ca^2+^/CaM–Ca_v_1.2-IQ_1665-1685_ interaction with Ca_v_1.2-NSCaTE_51-67_. Data were processed using the MicroCal PEAQ-ITC software. *K*_d_, binding affinity; N, stoichiometry; n, number of experimental replicates. The sum of the change in enthalpy (ΔH) and the change in entropy (ΔS) multiplied by the absolute temperature (T) gives the change in free energy (ΔG). Experiments were performed in the presence of 5 mM CaCl_2_ at 25 °C. Data are mean ± s.e.m. For comparison of affinity values, differences between groups were determined using two-tailed unpaired Student *t* test. For comparison of thermodynamic profiles, differences between groups were determined using a two-way ANOVA with Sidak’s multiple comparisons test. *p*-values are represented by stars with ∗∗∗*p* < 0.001 and ∗∗∗∗*p* < 0.0001. The ANOVA parameters are shown in Tables S9 and S10. DP, differential power; LQTS, Long QT syndrome; CaM, calmodulin; ITC, isothermal titration calorimetry.

Using ITC, we showed that Ca^2+^/CaM can bind at multiple sites on the Ca_v_1.2 channel. Through its N-lobe, CaM can interact with the N-terminal spatial Ca^2+^ transforming element (Ca_v_1.2-NSCaTE_51-67_) and through the C-lobe with residues from the IQ domain (Ca_v_1.2-IQ_1665-1685_) (59, 60). The stoichiometry of interaction of CaM with the binding motifs was measured as N∼2 for Ca_v_1.2-NSCaTE_51-67_ and N∼1 for Ca_v_1.2-IQ_1665-1685_ (Fig. 5, *A* and *B*). The disease-associated CaM variant E140G affected the interaction with both Ca_v_1.2-binding domains. The *K*_d_ was significantly increased from 1.08 ± 0.01 μM (WT, n = 5) to 8.33 ± 0.47 μM (E140G, n = 5) for Ca_v_1.2-NSCaTE_51-67_. Interestingly, the interaction between the E140G variant and Ca_v_1.2-IQ_1665-1685_ showed a significantly stronger binding affinity (∼2 fold) than WT (Fig. 5*C*). The thermodynamic parameters showed that the interaction of Ca^2+^/CaM-WT and E140G variant with Ca_v_1.2-binding sites was exothermic and driven by enthalpic contributions (Fig. 5*D*). The E140G variant showed a significant increase in enthalpy upon binding to Ca_v_1.2-NSCaTE_51-67_ domain, when compared to WT.

Physiologically, in the full Ca_v_1.2 channel complex, CaM is preassociated with higher affinity binding domains such as IQ. Therefore, we investigated the interaction between preassociated Ca^2+^/CaM-Ca_v_1.2-IQ_1665-1685_ and Ca_v_1.2-NSCaTE_51-67_ (Fig. 5*E*). In these conditions, the measured stoichiometry was N∼1 and the *K*_d_ was significantly increased from 2.23 ± 0.31 μM (WT, n = 5) to 25.2 ± 0.7 μM (E140G, n = 3). The interaction of Ca^2+^/CaM-Ca_v_1.2-IQ_1665-1685_ with Ca_v_1.2-NSCaTE_51-67_ was exothermic and driven by enthalpic contributions, with the E140G variant showing a significant increase in enthalpy upon binding.

### CaM-E140G does not alter RyR2-mediated Ca^2+^ release in cells

Using ITC, we showed that CaM can bind to the RyR2_3581-3608_ peptide in the absence and in the presence of Ca^2+^ with a stoichiometry of 1 (Fig. S2, *A* and *B*). The *K*_d_ of CaM for RyR2 is decreased from 2.59 ± 0.13 μM (n = 9) to 0.130 ± 0.001 μM (n = 6) upon Ca^2+^ binding, indicating a ∼20-fold stronger binding at saturating Ca^2+^ concentrations. The affinity of the E140G variant for RyR2_3581-3608_ remained unchanged in the absence of Ca^2+^ (*K*_d_ of 3.50 ± 0.55 μM, n = 5), however the affinity was decreased when Ca^2+^ was present (*K*_d_ of 0.254 ± 0.012 μM, n = 6) (Fig. S2*C*). In apo condition, the binding of RyR2_3581-3608_ to CaM variants was endothermic and entropy driven, whereas in the presence of Ca^2+^, the interaction was exothermic and enthalpy driven (Fig. S2*D*). In both Ca^2+^-free and Ca^2+^-bound conditions, the E140G mutant significantly increased the thermodynamic drive of the reaction, when compared to WT.

To determine the functional effect of E140G on Ca^2+^ release from the endoplasmic reticulum, human RyR2 and CaM variants were transiently overexpressed in HEK293T cells. In these experiments, CaM variants and the fluorescent marker (dTomato) were coexpressed under the control of the same promoter, as two distinct proteins and not as fusion proteins. Spontaneous Ca^2+^ oscillations were measured using Calbryte 520-AM (AAT Bioquest) as a Ca^2+^ indicator and single-cell fluorescence confocal microscopy (Fig. S3). Analysis of the kinetic parameters (Fiji and SignalFind) showed that LQTS-associated CaM mutation E140G did not significantly affect amplitude, rise and decay time, duration, inter-transient interval, or frequency of RyR2-mediated Ca^2+^ release events compared to CaM-WT.

### CaM-E140G has a reduced binding affinity for Ca^2+^ and altered secondary structures

**Figure 6:**
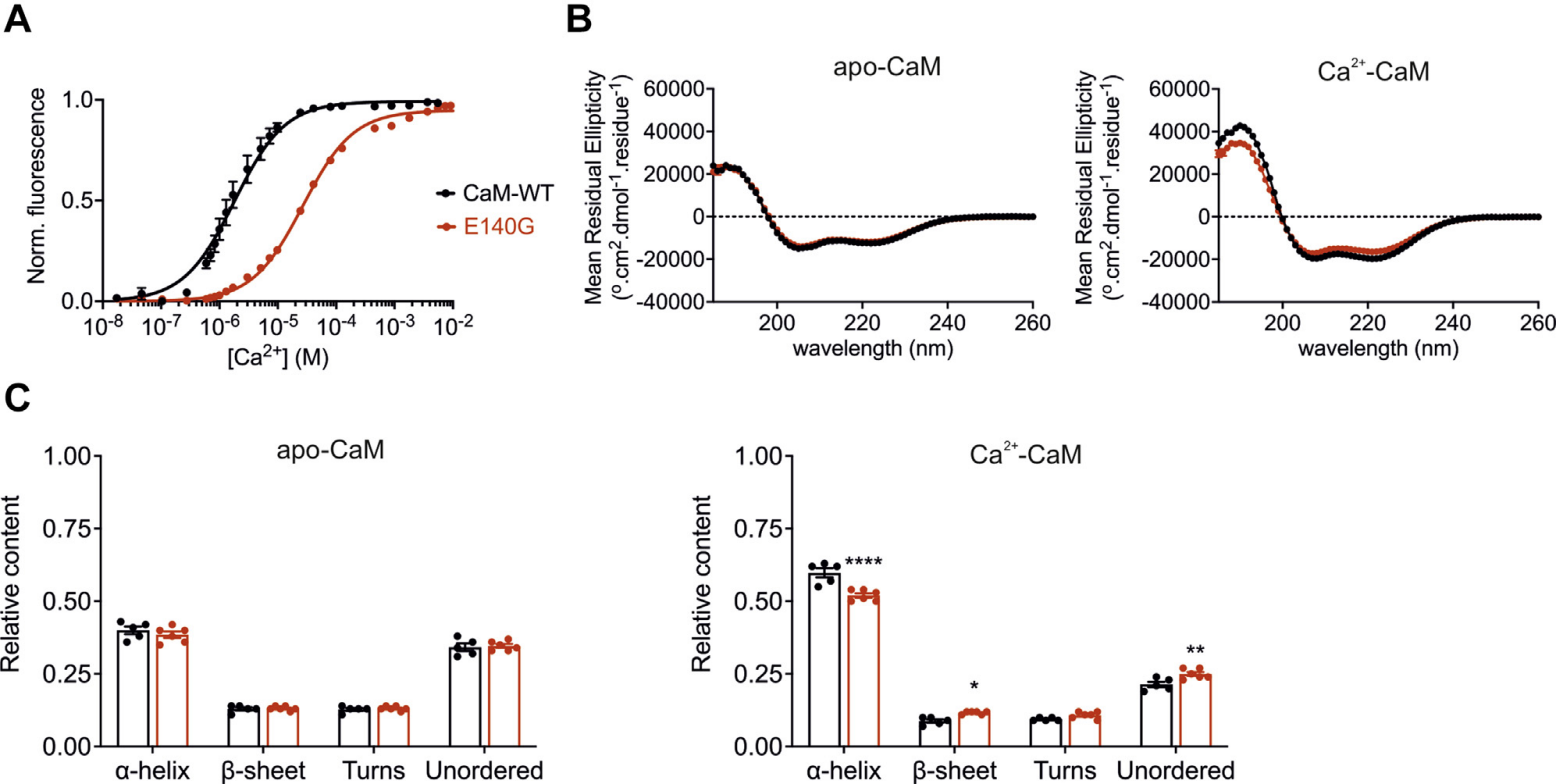
**E140G mutation decrease Ca**^**2+**^**-binding affinity and alter CaM secondary structure content.***A*, equilibrium Ca^2+^-binding titrations for CaM-WT and E140G measured by intrinsic tyrosine fluorescence (C-lobe specific) at 20 °C. Proteins (6 μM) were titrated with increasing free Ca^2+^ concentrations. Fluorescence changes (λ_ex_ = 277 nm and λ_em_ = 307 nm) are normalized to *F*_0_ of 0 and *F*_max_ of 1 and fitted to the Hill equation. Fitted curves are represented by solid lines overlaying the data points. Titrations were performed in five replicates and represented as mean ± s.e.m. *B*, circular dichroism spectra were obtained in the presence of 1 mM EGTA (*left panel*) or 1 mM CaCl_2_ (*right panel*). Data displayed are average traces for CaM-WT (n = 5) and CaM-E140G (n = 6). *C*, protein secondary structure content estimated using the CDSSTR algorithm (DichroWeb, reference set 7) in the presence of 1 mM EGTA (*left panel*) or 1 mM CaCl_2_ (*right panel*). Data are mean ± s.e.m. Experiments were performed at 20 °C, CaM-WT (n = 5) and CaM-E140G (n = 6). Differences between groups were determined using a two-way ANOVA with Sidak’s multiple comparisons test. *p*-values are represented by stars with ∗*p* < 0.05, ∗∗*p* < 0.01 and ∗∗∗∗*p* < 0.0001. The ANOVA parameters are shown in Table S11. CaM, calmodulin.

Equilibrium Ca^2+^-binding titrations were performed using intrinsic tyrosine fluorescence to determine the effect of E140G mutation on the interaction between Ca^2+^ and the C-lobe of CaM (Fig. 6*A*). Free Ca^2+^ concentrations ([Ca^2+^]) were calculated using the Maxchelator Web Maxc standard (61) program and were verified using the Ca^2+^ dye Cal520-FF (AAT Bioquest). Ca^2+^ titrations were normalized and fitted to Hill specific binding model to obtain the dissociation constant (K_d_). *K*_d_ was significantly increased from 1.65 ± 0.08 μM (CaM-WT, n = 5) to 28.3 ± 0.9 μM (CaM-E140G, n = 5), indicating a ∼17-fold decrease in Ca^2+^-binding affinity for the LQTS-associated variant. Cooperativity of binding (Hill coefficient) was reduced from 1.3 ± 0.1 (CaM-WT) to 0.9 ± 0.1 (E140G).

Secondary structure content was investigated by far-UV CD spectra analysis *via* the DichroWeb online server (Fig. 6, *B* and *C*). In the absence of Ca^2+^ (apo conditions), the secondary structure content of CaM-WT and the E140G variant were similar, both comprising of ∼40% of α-helices and 13% of β-sheets. When Ca^2+^ was present, we observed the characteristic increase in α-helical content for both variants, to 60 ± 2% for CaM-WT (n = 5) and 52 ± 1% for E140G (n = 5). Upon Ca^2+^ binding, CaM-E140G variant showed significantly lower α-helical content and more unordered structures, when compared to the WT protein (Fig. 6*C*).

### Protein susceptibility to temperature and protease digestion is affected by the LQTS-associated mutation E140G

To determine whether the LQTS-associated mutation E140G induced significant 3D conformational changes to CaM, we investigated WT and mutant CaM’s susceptibility to temperature and protease digestion (trypsin or the endoproteinase AspN) (Fig. S4). Using CD, we monitored protein unfolding as temperature increases for Ca^2+^-free (apo-) CaM proteins. We found that the E140G mutation did not significantly affect the melting temperature (Tm) with a Tm of 42.3 ± 0.5 °C for CaM-WT (n = 8) and 42.0 ± 0.2 °C for CaM-E140G (n = 6) (Fig. S4*A*). In addition, we determined the stability of CaM proteins using trypsin and AspN-limited proteolysis (Fig. S4, *B* and *C*). In the absence of Ca^2+^, 5 ng/μl trypsin and 0.5 ng/μl AspN were sufficient to achieve full protein degradation. We did not observe any significant difference in protease susceptibility for CaM-WT and E140G. When Ca^2+^ is present, CaM proteins are significantly more resistant to degradation with 4-fold more trypsin (20 ng/μl) required to achieve full degradation, while AspN did not completely digest the proteins at the concentrations tested. For Ca^2+^-bound proteins, we observed that E140G was significantly more sensitive to both trypsin and AspN cleavage (Fig. S4, *B* and *C*).

Altogether, these data suggest that the disease-associated mutation E140G may affect the 3D structure of the protein and the exposure of specific residues involved in protease digestion.

## Discussion

CaM is a major Ca^2+^ sensor that regulates several target proteins involved in cardiac muscle contraction, including RyR2, Ca_v_1.2, and CaMKIIδ (25, 27, 36, 46, 62, 63, 64, 65, 66, 67). Tightly controlled CaM-target interactions are crucial for maintaining Ca^2+^ homeostasis and cardiovascular function. Perturbed interactions between CaM and proteins involved in cardiac muscle contraction, resulting from genetic mutations can cause severe cardiac syndromes including LQTS (reviewed by (7, 68)). In this article, we investigated the effect of CaM-E140G, a human variant associated with LQTS, on the structure-function relationship of CaM. In a whole genome sequencing study of elusive LQTS cases, Boczek *et al.* identified six CaM missense variants including E140G (c.422 A > G, *CALM1*) (11). CaM is a highly conserved Ca^2+^-sensing protein encoded by three independent genes *CALM1*, *CALM2*, and *CALM3*, and all three encode an identical protein (69, 70). The heterozygous variant E140G only affects 1/6 of the CaM alleles and still results in life-threatening arrhythmia, demonstrating the dominant effect of the mutation. The CaM-E140G variant is a *de novo* mutation found in an Indian male who experienced his first syncope event at 3 years old and his first sudden cardiac arrest at 4 years old. The patient was treated with beta-blockers and sodium channel blockers, but his QTc interval remained unchanged (610 ms) and he is presenting speech and motor skill delay.

Using a combination of electrophysiology, cell biology, biophysics, and structural biology techniques, we present novel data showing that the disease-associated mutation E140G has a significant effect on key functional properties of CaM, including the regulation of important targets such as CaMKIIδ and Ca_v_1.2. Table 2 and Table 3 summarize the major biophysical and functional properties of LQTS-associated CaM variant E140G determined in this study, respectively.

**Table 2:** Summary of binding affinities of LQTS-associated CaM variant E140G for Ca^2+^, Ca_v_1.2, RyR2, and CaMKIIδ peptides, compared to CaM-WT

CaM variant	Ca^2+^	Ca_v_1.2			RyR2		CaMKIIδ
		(NSCaTE)	(IQ)	(prebound-IQ)	(apo)	(Ca^2+^)	
CaM-WT	1.65 ± 0.08	1.08 ± 0.01	0.030 ± 0.001	2.23 ± 0.31	2.59 ± 0.13	0.130 ± 0.001	0.052 ± 0.008
E140G	28.3 ± 0.9∗∗∗∗	8.33 ± 0.47∗∗∗∗	0.014 ± 0.002∗∗∗	25.2 ± 0.7∗∗∗∗	3.50 ± 0.55	0.254 ± 0.012∗∗∗∗	0.097 ± 0.008∗∗

*K*_d_ values (μM), mean±s.e.m.

∗∗*p* < 0.01.

∗∗∗∗*p* < 0.0001 *versus* CaM-WT.

**Table 3:** Summary of functional effects of LQTS-associated CaM variant E140G on CaMKIIδ and ion channel activity

CaM variant	CaMKIIδ (biochemical assays)		Ca_v_1.2 (patch-clamp electrophysiology)				RyR2 (calcium imaging)		
	Syntide2 (a.u.)	Phospho-Thr287 (5 min, a.u.)	Peak current density (pA/PF)	V_50_ activation (mV)	V_50_ inactivation (mV)	CDI (f300)	Frequency (events/min)	Duration (seconds)	Amplitude (Δ*F*)

CaM-WT	100.0 ± 2.6	0.30 ± 0.05	−5.17 ± 0.60	4.52 ± 2.11	−19.71 ± 2.22	0.76 ± 0.05	2.8 ± 0.9	10.5 ± 0.9	25.7 ± 7.0
E140G	65.7 ± 5.4∗∗∗∗	0.01 ± 0.008∗∗	−3.50 ± 0.43	3.59 ± 1.98	−14.55 ± 1.84	0.18 ± 0.04∗∗∗∗	3.1 ± 0.2	10.9 ± 0.4	30.8 ± 4.5

Values are mean ± s.e.m.

∗∗*p* < 0.01.

∗∗∗∗*p* < 0.0001 *versus* CaM-WT.

In many cases, disease-associated variants can affect protein folding and/or intracellular protein stability. We showed that the secondary structure content, susceptibility to protease digestion and thermostability of apo-CaM, was not affected by the E140G mutation. However, we observed that upon Ca^2+^ binding, Ca^2+^/CaM-E140G had a significantly lower α-helical content and was more susceptible to protease digestion than Ca^2+^/CaM-WT. Through its four EF-hand motifs, CaM can sense intracellular Ca^2+^ across a wide range of concentrations to trigger the appropriate functional outcome. Mutation in the residues at the EF-hands involved in direct binding with the Ca^2+^ ions can result in severe consequences. E140 is one of the residues that directly participate in Ca^2+^ binding at the C-terminal EF hand 4 of Ca^2+^/CaM. Our equilibrium Ca^2+^-binding titrations data showed a ∼17-fold decrease in Ca^2+^ affinity for the CaM-E140G variant compared to CaM-WT, which is in accordance with previous studies (11). In addition, we observed that cooperativity of binding to Ca^2+^ was significantly reduced for E140G when compared to CaM-WT. A loss of Ca^2+^ affinity and cooperativity have been shown for other arrhythmia-associated CaM variants (71).

Altogether, these data suggest a defect in Ca^2+^ sensing associated with an improper transition from the Ca^2+^-free to the Ca^2+^-bound conformation for the E140G variant. In all our *in vitro* experiments, a super saturating concentration of Ca^2+^ (5 mM CaCl_2_) was added to eliminate any effect arising due to impaired Ca^2+^ binding.

CaM can modulate protein activity relevant to cardiac muscle contraction *via* phosphorylation. One of the major kinases involved in RyR2 and Ca_v_1.2 channel regulation is CaMKIIδ (64, 72, 73, 74, 75, 76). CaMKIIδ is a multimeric Ser/Thr protein kinase which, upon Ca^2+^/CaM activation, will autophosphorylate and then phosphorylate target channels to regulate their function. Using the genetically encoded CaMKII sensor ‘Camui’, Hwang *et al.* showed that CaMKII binding and activity remained unchanged for LQTS-associated CaM mutants D95V, D129G, F141L (77). However, another research group showed a decreased activation of CaMKII in the presence of CaM variants N97S, D95V, and D129G (78). We previously investigated CPVT-associated CaM variants and showed that CaM-N53I did not affect the kinase activity of CaMKIIδ, whereas CaM-A102V significantly increased substrate phosphorylation levels by ∼60% (79). Interestingly, autophosphorylation levels between CaM-WT and CPVT-associated mutants were not significantly different, demonstrating that the increase in kinase activity observed for CaM-A102V cannot be attributed to enhanced autophosphorylation. Therefore, information on the effect of arrhythmia-associated CaM mutations on CaMKIIδ activity is limited and controversial. In our study, we present novel data showing that Ca^2+^/CaM-E140G significantly decreased the substrate phosphorylation levels from CaMKIIδ by ∼35%, when compared to WT. Further, we demonstrated for the first time that the decrease in kinase activity for E140G could be attributed to impaired CaMKIIδ autophosphorylation. We showed that the binding affinity of Ca^2+^/CaM for CaMKIIδ_294-315_ was significantly decreased for the E140G variant and obtained the first high-resolution structure of Ca^2+^/CaM-CaMKIIδ_294-315_ for an LQTS variant. Upon structural superimposition, we observed significant differences at the CaM N-terminal region despite the E140G mutation being at the C-terminal region, suggesting a global structural rearrangement. The H-bond and salt bridge interactions between CaM and CaMKIIδ_294-315_ showed subtle variations between the CaM-WT and CaM-E140G variant. The additional H-bonds observed for the E140G mutant support the increased ΔH contribution measured by ITC. Compared to CaM-WT, the variant E140G showed a missing H-bond at Lys75[NZ]:Met15[O]. This structural discrepancy is likely responsible for the missing α-helix between Phe65 and Lys77. CaMKIIδ_294-315_ peptide residues showing electron density maintained all the salt-bridge interaction in the CaM-E140G complex, however residues and individual atoms from CaM involved in these interactions are different, especially with residues from the CaM N-terminal region. Considering that [CaM]_free_ in a cardiomyocyte is relatively low, estimated to be around 50 to 75 nM (compared to ∼6 μM [CaM]_total_) (80), a 2-fold reduction in affinity alone may only have limited relevance *in vivo*. However, we believe that the combination of impaired binding of CaM-E140G to CaMKIIδ_294-315_ and the altered 2D-3D structure of CaM-E140G underlies the autophosphorylation defect of the kinase.

During excitation-contraction coupling, Ca^2+^ enters the cell through voltage-gated Ca^2+^ channels (Ca_v_1.2) to trigger intracellular signaling cascades leading to cardiac muscle contraction. CaM can regulate the activity of Ca_v_1.2 in a Ca^2+^-dependent manner (81). CaM can inactivate and activate Ca_v_1.2 channels depending on the cytosolic Ca^2+^ concentration (22, 23, 24). In fact, CaM plays a central role in CDI, a physiologically important negative feedback process to regulate intracellular Ca^2+^ concentrations. Using a HEK293 cellular model expressing Ca_v_1.2 channel, we observed no significant change in the voltage-dependence of activation and inactivation of Ca_v_1.2. Importantly, our use of Ba^2+^ allowed us to resolve Ca^2+^-dependent and Ca^2+^-independent inactivation and provided a clearer insight into the mechanisms of E140G dysregulation of Ca_v_1.2. We determined the first time the precise contribution of CDI (compared to voltage-dependent inactivation) in the inactivation of the channel. We demonstrated a significant reduction in the CDI of Ca_v_1.2 when CaM-E140G was present, which could be the result of a combination of defective Ca^2+^ sensing and altered interaction with the channel binding domains. This observation is in line with previously published data on LQTS-associated CaM mutants, implying a potential common mechanism of disease involving disrupted Ca_v_1.2 inactivation (11, 14, 18, 82, 83). The defect in Ca_v_1.2 inactivation will elongate the ventricular action potential (AP), which would result in an increase of the QT interval, characteristic of LQTS. Interestingly, our cellular model still expressed endogenous CaM (as it would be in human patients), demonstrating that E140G detrimentally impacted Ca_v_1.2 CDI even in the presence of CaM-WT. This is in accordance with a previous study which used a 1:3 ratio of CaM-E140G:CaM-WT protein introduced into murine ventricular cardiomyocytes where they observed a significant increase in I_Ca_ rate of inactivation (11). These data showed that even as the nondominant isoform, CaM-E140G can detrimentally affect Ca_v_1.2 inactivation and produces a dominant disease phenotype.

The precise molecular mechanism of CaM-induced inactivation of the channel is still elusive. At the C-terminal, CaM can bind at regions such as IQ and pre IQ (C and A) (60, 65, 84, 85) with CaM C-lobe showing strongest affinity towards IQ domain (86, 87). At the N-terminal, CaM binds at NSCaTE region (86, 88, 89). The CaM N-lobe was shown to have a stronger affinity towards NSCaTE than the C-lobe interaction (88). However, an overall picture of how CaM modulates the activity of the channel through interaction with all of these domains is unclear. We showed that Ca^2+^/CaM-WT and Ca^2+^/CaM-E140G variants had energetically favorable binding to Ca_v_1.2 channel peptides as indicated by negative ΔG. These interactions were predominantly enthalpy-driven, suggesting that binding mainly consisted of hydrogen bond formation. Interestingly, we observed that the E140G mutation significantly affected the thermodynamics of binding (ΔH and ΔS) to Ca_v_1.2-binding domains, suggesting subtle changes in the mechanism of interaction, when compared to CaM-WT. We observed a binding stoichiometry (N) of ∼2 for the Ca^2+^/CaM–Ca_v_1.2-NSCaTE_51-67_ interaction which was in accordance with several studies (86, 88), in contrast to Benmocha *et al.* who reported a stoichiometry of N = 1 (89). A stoichiometry of N∼2 implies the possibility of both the N lobe and C lobe of CaM interaction with Ca_v_1.2-NSCaTE. This observation is unlikely to be physiologically relevant, as *in vivo*, CaM would be preassociated to higher affinity binding domains (such as Ca_v_1.2-IQ); therefore, CaM would present a stoichiometry of binding with Ca_v_1.2–NSCaTE of only N∼1. For Ca^2+^/CaM–Ca_v_1.2-IQ_1665-1685_ binding events, we measured a binding stoichiometry of ∼1, as shown in previous studies (86, 87). Using ITC, we measured the interaction between preassociated Ca^2+^/CaM–Ca_v_1.2-IQ_1665-1685_ and Cav1.2-NSCaTE_51-67_. In these more physiological conditions, we obtained a stoichiometry of N∼1. This is consistent with only one lobe of CaM binding to Ca_v_1.2-NSCaTE, in accordance with previous studies (86).

Based on our ITC data, the stoichiometry of binding of CaM with Ca_v_1.2 peptides is not affected by the LQTS-associated mutation E140G. For Ca^2+^/CaM–Ca_v_1.2-NSCaTE_51-67_, we measured a binding affinity of 1.08 ± 0.01 μM, which was in the same range as previously reported (*K*_d_ = 0.57–2.90 μM) (86, 88, 89). The E140G variant significantly increased the dissociation constant with a *K*_d_ of 8.33 ± 0.47 μM. Interestingly, when CaM is preassociated with Ca_v_1.2-IQ_1665-1685_, we demonstrated that the *K*_d_ for Cav1.2-NSCaTE_51-67_ is still significantly increased for the LQTS-associated variant, from 2.23 μM (CaM-WT) to 25.2 μM (CaM-E140G). These data suggest that the E140G mutation, while located in the C-lobe, induces global structural rearrangements which reduces CaM binding to NSCaTE and therefore could contribute to defects in Ca_v_1.2 CDI. We measured a 2-fold increase in affinity for Ca^2+^/CaM-E140G–Ca_v_1.2-IQ_1665-1685_ when compared to WT, which differs from the ∼3-fold reduction in affinity previously observed by GST-pull down assay (82). However, using HEK293 cells and a FRET biosensor, Limpitikul *et al.* also observed an increase in binding affinity of LQTS-associated CaM variants (e. g. D95V, F141L) for the pre-IQ/IQ domain (83).

Several studies have emphasized the importance of CaM binding with the Ca_v_1.2 IQ domain for regulating channel inactivation (CDI). It has been established that a single CaM prebound to the Ca_v_1.2-IQ domain is necessary and sufficient to produce CDI of its associated channel (90, 91). Mutations on CaM or IQ domain which weaken or abolish this interaction have been shown to affect CDI (65, 91, 92, 93). These data suggest a unique mechanism for E140G where the LQTS-associated variant can outcompete CaM-WT for binding to the C-terminal IQ domain of the channel, therefore preventing appropriate downstream regulation of Ca_v_1.2 CDI through the NSCaTE domain. For Ca_v_1.2, CaMKII-induced phosphorylation is essential for Ca^2+^-dependent I_Ca_ facilitation (76, 94). Impaired binding of the CaM-E140G variant to Ca_v_1.2 channel peptides along with reduced phosphorylation activity of CaMKIIδ in the presence of CaM-E140G variant points towards a possibility of disrupted CDF as well as CDI. In addition, impaired CaM regulation of CaMKIIδ could affect other components of cardiomyocyte excitability such as voltage-gated Na^+^ and K^+^ channels and phospholamban.

As part of the excitation-contraction coupling process, Ca^2+^ entering the cell through Ca_v_1.2 binds to the RyR2 to activate Ca^2+^ release from the SR. Ca^2+^ will then diffuse to the myofibrils to generate muscle contraction. CaM is known to bind and regulate the open probability of the RyR2 channel (95). Using ITC, we determined that the E140G mutation did not affect the binding of apo-CaM to RyR2_3581-3608_, while it decreased the affinity in the presence of saturating Ca^2+^ concentrations. These observations differ from Søndergaard *et al.* (95) who, using fluorescence anisotropy-based affinity measurement, saw an increase in the binding affinity for apo-CaM-E140G with RyR2 and no change in affinity in high Ca^2+^ conditions. However, we observed using confocal imaging that the reduced affinity of E140G for RyR2 was not sufficient to alter RyR2 Ca^2+^ release dynamics, as previously observed (11).

In addition, RyR2 needs to be phosphorylated either at S2808 by PKA or at S2814 by CaMKIIδ for CaM to exert its inhibitory effect (46). Since the phosphorylation can be compensated by PKA, the reduced activity of CaMKIIδ when E140G is present may not have a direct effect on Ca^2+^ cycling homeostasis through RyR2 on the SR, as observed in our Ca^2+^ imaging experiments. Interestingly, approximately 20% of the total CaM expressed within the cell is bound to RyR2 (39). Since the CaM-E140G variant has a decreased affinity towards RyR2, the excess unbound CaM is free to bind to other targets. The increased likelihood for CaM-E140G to interact with the Ca_v_1.2 channel and CaMKIIδ would promote loss of CDI and elongate the cardiac AP which would increase the QT interval.

**Figure 7:**
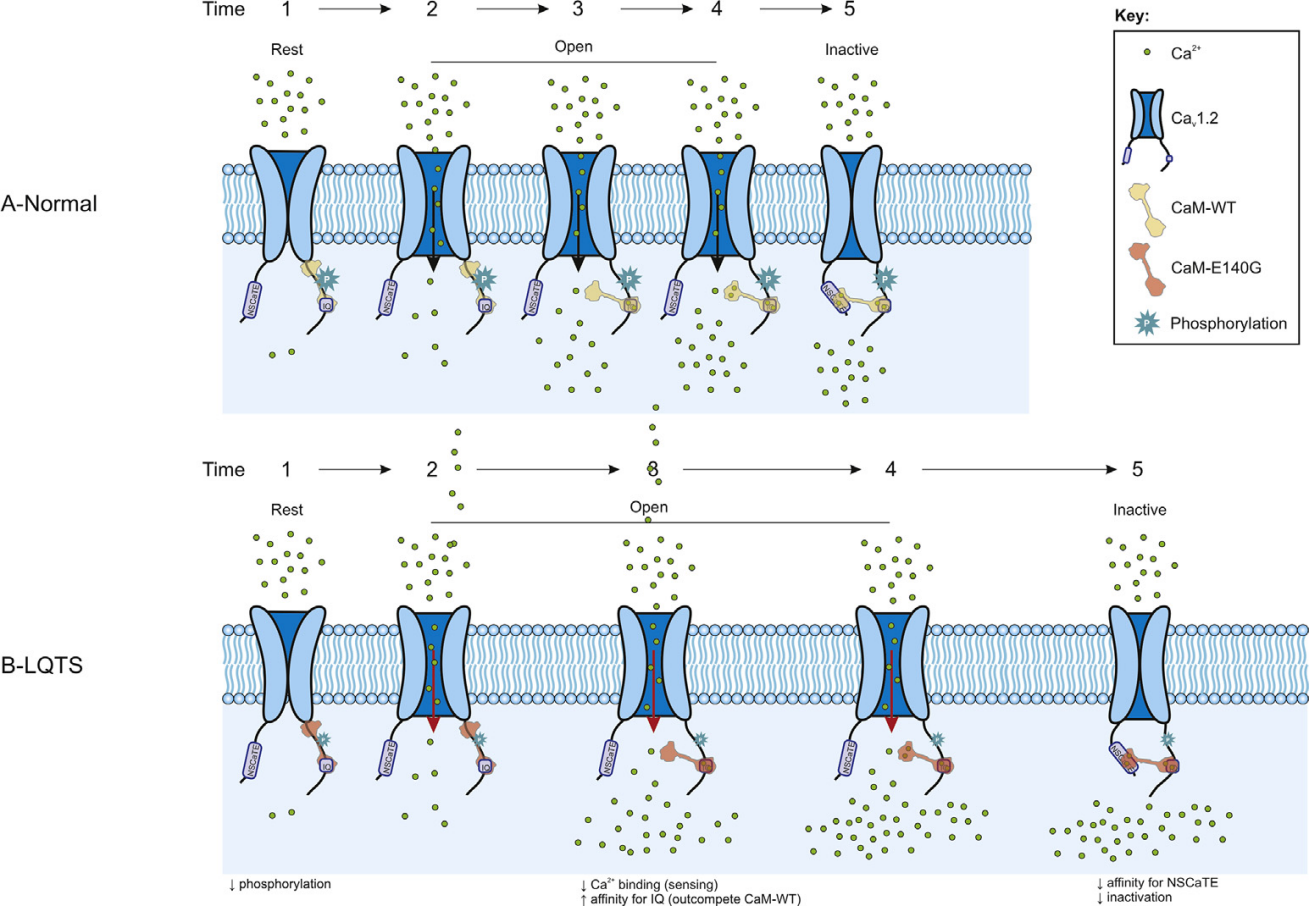
**Proposed regulatory mechanism for LQTS-associated CaM variant E140G.** A-Normal. At rest, CaM is bound to the intracellular IQ region of Ca_v_1.2, close to the opening of the channel pore, where it is ideally placed to monitor Ca^2+^ influx into the cytosol (Time 1). Upon stimulation (action potential), the Ca_v_1.2 channel opens and Ca^2+^ enters into the cardiomyocyte (Time 2–4). CaM can sense Ca^2+^ influx and when intracellular [Ca^2+^] increases, CaM binds to the Ca_v_1.2 NSCaTE domain to initiate the inactivation of the channel (Time 4–5). This prevents further Ca^2+^ influx and protect the cell from Ca^2+^ overload. B-LQTS. In LQTS, CaM-E140G affinity for Ca^2+^ is reduced, therefore intracellular [Ca^2+^] changes are not sensed appropriately and Ca_v_1.2 remains open (Time 2–4 is longer). Additionally, at elevated intracellular [Ca^2+^], CaM-E140G has a higher affinity for Ca_v_1.2-IQ, suggesting it may outcompete CaM-WT on the C-terminal domain of the channel (Time 3). This is particularly important as CaM-E140G has a reduced binding affinity for the NSCaTE domain, which will impair CaM-dependent CDI (Time 4–5 longer). In addition, CaMKIIδ bound to CaM-E140G shows significantly reduced kinase activity, which will reduce phosphorylation of Ca_v_1.2. As CaMKIIδ-mediated channel phosphorylation is required for CaM to bind to Ca_v_1.2, reduced CaMKIIδ activity will also affect CaM-dependent CDI. Altogether, *via* a complex mechanism involving impaired Ca^2+^ sensing, CaMKIIδ activation and Ca_v_1.2 CDI, CaM-E140G would promote dysregulation of Ca^2+^ homeostasis and the prolonged AP duration characteristic of LQTS. AP, action potential; CaM, calmodulin; CDI, Ca^2+^-dependent inactivation; LQTS, Long QT syndrome.

In summary, the LQTS-associated mutation E140G affects important cellular functions of CaM. We demonstrate that reduced CaMKIIδ phosphorylation and impaired Ca_v_1.2 CDI are key parameters involved in the molecular aetiology of the disease, both contributing to prolonged Ca^2+^ influx and an increased depolarizing drive that elongates the ventricular AP (Fig. 7).

Interestingly, Hegyi *et al.* (2021) showed that inhibition of CaMKII (using AIP) has no effect on the AP in an ATX-II model of LQTS (96). ATX-II is a neurotoxin which delays sodium channel inactivation and therefore is likely to involve distinct molecular pathways, independent from CaM or CaMKII activity. In addition, they performed experiments in a CaM-WT background, whereas our study focused on CaM-E140G. Because Ca_v_1.2 phosphorylation is required for CaM binding (97), we believe that reduced phosphorylation activity when CaM-E140G is present would decrease CaM binding to the channel and therefore reduce Ca_v_1.2 CDI. This would result in an elongation of the AP and account for the clinical presentation in CaM-E140G patients.

CaM is bound to the intracellular IQ region of Ca_v_1.2, close to the opening of the channel pore, where it is ideally placed to monitor Ca^2+^ influx into the cytosol (60). Mutated CaM-E140G affects Ca_v_1.2 CDI in two key ways: 1) CaM-E140G affinity for Ca^2+^ is reduced, therefore intracellular [Ca^2+^] changes are not sensed appropriately and 2) CaM-E140G has a reduced binding affinity for NSCaTE, the intracellular channel domain that Ca^2+^-bound CaM needs to dock onto to induce CDI. Additionally, at elevated intracellular [Ca^2+^], CaM-E140G has a higher affinity for Ca_v_1.2-IQ, suggesting it may outcompete CaM-WT on the C-terminal domain of the channel. In combination, these alterations will severely impair CaM-dependent CDI. Importantly, we also show that CaMKIIδ activity is affected by CaM-E140G, adding another layer of complexity. CaMKIIδ bound to CaM-E140G shows significantly reduced kinase activity, which will reduce the phosphorylation of Ca_v_1.2. As CaMKIIδ-mediated channel phosphorylation is required for CaM to bind to Ca_v_1.2 (97), reduced CaMKIIδ activity will also affect CaM-dependent CDI.

Altogether, *via* a complex mechanism involving impaired Ca^2+^ sensing, CaMKIIδ activation, and Ca_v_1.2 CDI, CaM-E140G would promote the dysregulation of Ca^2+^ homeostasis and the prolonged AP duration characteristic of LQTS. Our data in particular highlights the novel effects of CaM-E140G on the activity of CaMKIIδ, an enzyme often overlooked in the development of LQTS and a potential future therapeutic target.

## Experimental procedures

### Plasmids

For the biophysical experiments, recombinant protein was obtained by cloning the human CaM gene into pE-SUMOPro as previously described (79). CaM variant E140G was generated by site-directed mutagenesis following the QuikChange protocol (Agilent Technologies). The primers used were forward 5′-GATGGTCAAGTAAACTATGAAGGGTTTGTACAAATGATGACAGCA-3′ and reverse: 5′-TGCTGTCATCATTTGTACAAACCCTTCATAGTTTACTTGACCATC-3’.

For the cellular experiments, CaM WT and E140G were subcloned from pE-SUMOPro (described above) into pHIV-IRES-dTomato or pHIV-IRES-EGFP (Addgene plasmid 21374, 21373, gifts from Bryan Welm) using Gibson Assembly as follows. The primers used to linearize the pHIV-IRES-dTomato or pHIV-IRES-EGFP backbone were forward 5′-AGGATCCGCCCCTCTCCC-3′ and reverse 5′-CGCTCACGACACCTGAAATG-3’; the primers to amplify the CaM genes were forward 5′-CATTTCAGGTGTCGTGAGCGATGGCTGACCAACTGACTG-3′ and reverse 5′-GAGGGAGAGGGGCGGATCCTTCACTTTGCTGTCATCATTTG-3’. PCR was performed using Phusion High-Fidelity DNA Polymerase (New England Biolabs). Fragments were assembled using the NEBuilder HiFi assembly kit according to the manufacturer's recommendations to generate pHIV-CaM-IRES-dTomato or pHIV-CaM-IRES-EGFP. In these constructs, CaM variants and the fluorescent marker (dTomato or EGFP) were coexpressed under the control of the same promoter, as two distinct proteins and not as fusion proteins. pHIV-CaM-IRES-dTomato was used in the Ca^2+^-imaging experiments, whereas the pHIV-CaM-IRES-EGFP was used for patch-clamp electrophysiology.

The molecular construct for expressing the human RyR2 (pcDNA-3/eGFP-hRyR2) was a kind gift from Dr Chris George (Swansea University Medical School).

All resulting plasmids were confirmed by DNA sequencing (DNA Sequencing and Services, University of Dundee).

### Peptides

The peptides encompassing CaM-binding domains in CaMKIIδ, Ca_v_1.2, and RyR2 as well as syntide2 (CaMKIIδ substrate) were chemically synthesized, HPLC-purified, and purity was estimated as >95% through mass spectrometry (GenicBio).

CaMKIIδ_294-315_: FNARRKLKGAILTTMLATRNFS; Ca_v_1.2-NSCaTE_51-67_: SWQAAIDAARQAKLMGS; Ca_v_1.2-IQ_1665-1685_: KFYATFLIQEYFRKFKKRKEQ; RyR2_3581-3608_: RSKKAVWHKLLSKQRKRAVVACFRMAP; Syntide-2: PLARTLSVAGLPGKK (54, 59, 60, 98, 99).

### CaM recombinant protein expression and purification

CaM-WT and E140G variant were expressed and purified as previously described (79). In short, CaM pESUMOPro-Kan plasmids were transformed into *Escherichia coli* BL21(DE3) STAR and cultured in 2xYT media containing 100 μg/ml kanamycin. Expression was induced with 0.5 mM IPTG overnight at 18 °C. Cells were harvested by centrifugation and pellets were resuspended in 50 mM Hepes, 200 mM NaCl, pH 7.5 supplemented with protease inhibitor cocktail (Proteoloc, Abcam). Cells were lysed with lysozyme (1 mg/ml) for 30 min on ice followed by sonication. Lysates were further treated with BaseMuncher (Abcam) and clarified by ultracentrifugation.

Clarified lysates were purified on a HisTrap HP column (ÄKTA Start, GE Healthcare) using a linear gradient of 0 to 500 mM imidazole. Eluted proteins were dialyzed overnight at 4 °C (8 kDa) to remove the imidazole, and His-tag was removed by treatment with SUMO protease (ULP1). CaM proteins were then further purified by reverse HisTrap and size-exclusion chromatography (HiLoad Superdex 75pg, ÄKTA Pure, GE Healthcare). Fractions containing the purified proteins were concentrated using Amicon centrifugation units (3 kDa), flash-frozen in liquid nitrogen, and stored in -80 °C until used.

### Protein and peptide concentration measurements

Concentrations were obtained by measuring absorbance at 280 nm using a DS-11+ spectrophotometer (DeNovix) and molar extinction coefficients calculated from the amino acid composition (ExPASy/ProtParam program) (100). ε_0_ (CaM, Ca_v_1.2-IQ_1665-1685_) = 2980 M^−1^ cm^−1^; ε_0_ (Ca_v_1.2-NSCaTE_51-67_, RyR2_3581-3608_) = 5500 M^−1^ cm^−1^.

For CaMKIIδ_294-315_ and Syntide-2 peptides, because the amino acid sequence does not contain any tryptophan or tyrosine, concentrations were determined using the Pierce Quantitative Colorimetric Peptide Assay kit (Thermo Fisher Scientific) as per the manufacturer’s instruction.

### CaMKIIδ phosphorylation activity

#### Kinase activity

CaMKIIδ phosphorylation activity was determined using the Amplite universal fluorimetric kinase assay kit (AAT Bioquest) following the manufacturer’s recommendations. The kinase reaction contained 4 μM CaM protein, 0.03 μM GST-CaMKIIδ (Abcam), 100 μM Syntide-2 peptide substrate (Genicbio), 200 μM ATP, and 2 mM CaCl_2_. The mixture was incubated at room temperature for 20 min prior to the addition of the ADP sensor. The reaction mix was further incubated at room temperature for 20 min before measuring the fluorescence intensity on a FLUOstar Omega (BMG Labtech) at λ_ex_ 545 nm and λ_em_ 590 nm.

#### Autophosphorylation activity

CaM protein (1 μM) and GST-CaMKIIδ (300 nM) were incubated in 50 mM K^+^-Hepes, pH 7.5, 100 mM KCl, 2 mM MgCl_2_, 5 mM 1,4-DTT, 100 μM CaCl_2_ at room temperature. The reaction was started by adding 300 μM ATP and was terminated using SDS-containing sample buffer at predefined time points (0 min, 5 min, 15 min, 30 min, and 60 min). Post separation of proteins by SDS-PAGE (NuPage 4–12% Bis-Tris), proteins were electro-transferred to a nitrocellulose membrane using iBlot2 gel transfer device (7 min protocol consisting of three steps: 20 V for 1 min, 23 V for 4 min, and 25 V for 2 min). The membranes were blocked with 5% (w/v) fat-free powdered milk in 0.1% (v/v) Tween-20, 50 mM Tris–HCl (pH 7.6), 150 mM NaCl (T-TBS buffer). Membranes were then probed overnight at 4 °C with mouse anti-GST (Sigma-Aldrich, G1160) and rabbit anti-phospho T287 (Abcam, ab182647) monoclonal primary antibodies at 1/1000 and 1/500 dilutions, respectively. Next, membranes were washed in T-TBS and incubated for 1 h at room temperature with IRDye 680RD donkey anti-mouse (LI-COR, 926-68072) and IRDye 800CW donkey anti-rabbit (LI-COR, 926-32213) IgG secondary antibodies at 1/10,000 dilution. The bands were visualized using an Odyssey CLx infrared imaging system and the intensity of the bands were quantified by densitometry using Fiji (101).

### X-ray crystallography

High-throughput screening was performed using a Screenmaker crystallization robot (Innovadyne) with a final drop volume of 400 nl (protein:precipitant ratio of 1:1, reservoir volume 80 μl) and the sitting drop vapor diffusion method. Five commercial crystallization screens were used: JCSG+ (Molecular Dimensions), Morpheus (Molecular Dimensions), PEGRx (Hampton Research), Structure (Molecular Dimensions), and Wizard Classic (Molecular Dimensions). Crystals for Ca^2+^/CaM-WT–CaMKIIδ_294-315_ and Ca^2+^/CaM-E140G–CaMKIIδ_294-315_ were grown at 20 °C (1 mM CaM, 1 mM CaCl_2_, and 1.2 mM CaMKIIδ_294-315_ peptide). Ca^2+^/CaM–CaMKIIδ_294-315_ complexes crystallized using the following precipitant solutions: 0.2 M zinc acetate, 0.1 M imidazole, pH 8.0, and 18% w/v PEG 3000 for the WT; 0.1 M Na^+^-Hepes, 0.1 M MOPS (acid), pH 7.5, 0.03 M magnesium chloride hexahydrate, 0.03 M calcium chloride dihydrate, 12.5% v/v MPD; 12.5% PEG 1000; 12.5% w/v PEG 3350 for the E140G mutant.

For data collection, crystals were grown at 20 °C on a hanging drop format using 48-well plates with sealant (Hampton Research) and 12 mm siliconized glass circle coverslides (Hampton Research), with a final drop volume of 2 μl (protein:precipitant ratio of 1:1, reservoir volume 200 μl).

Crystals were cryo-protected with 25% (v/v) glycerol, and diffraction data were collected using the Diamond synchrotron beamline i04. Data were processed by xia2/DIALS or xia2/XDS/XSCALE (102, 103) and scaled with AIMLESS (104) in the CCP4i2 program suite (105). The structure for Ca^2+^/CaM-WT–CaMKIIδ_294-315_ was solved by molecular replacement with MOLREP (106), using PDB 2WEL as a search model (54) and refined using REFMAC (107). The structure of Ca^2+^/CaM-E140G–CaMKIIδ_294-315_ variant was refined starting from the WT structure. Rebuilding of the model between refinement cycles and adding water molecules was performed in COOT (108). The quality of the models was assessed on the MolProbity server (109).

A summary of diffraction data, refinement statistics, and the quality indicators for the structures are featured in Table 1. PDB codes are 7ZRP for Ca^2+^/CaM-WT–CaMKIIδ_294-315_ and 7ZRQ for Ca^2+^/CaM-E140G–CaMKIIδ_294-315_.

### Isothermal titration calorimetry

CaM proteins (WT or E140G) were used in the range of 10 to 50 μM and the peptides (CaMKIIδ_294-315_; Ca_v_1.2-NSCaTE_51-67_; Ca_v_1.2-IQ_1665-1685_; RyR2_3581-3608_) in 10- to 15-fold molar excess. For the preassociation experiments, CaM variants (100 μM) were mixed with an equimolar ratio of Ca_v_1.2-IQ_1665-1685_ (100 μM) and titrated against 5- to 10-fold molar excess of Ca_v_1.2-NSCaTE_51-67_.

Titrations were performed in 50 mM Hepes, 100 mM KCl, 2 mM MgCl_2_, pH 7.5 supplemented with either 5 mM CaCl_2_ (except for RyR2_3581-3608_ where either 5 mM CaCl_2_ or 5 mM EGTA was used to determine Ca^2+^-dependent and Ca^2+^-independent interactions, respectively). The peptides were titrated against CaM proteins across 20 injections (2 μl each) lasting 4 seconds with a 180 s grace period in-between.

All titrations were performed using a MicroCal iTC200 and automated PEAQ-ITC systems (Malvern Panalytical) at 25 °C under continuous stirring at 800 rpm. Data were processed using the MicroCal PEAQ-ITC software and fitted to a one-site binding model to derive the dissociation constant (*K*_d_), stoichiometry of binding (N), and thermodynamic parameters (Gibbs free energy ΔG, enthalpy change ΔH, and the entropic term ΔS).

### Whole-cell patch-clamp electrophysiology

#### Stable cell culture and transfection

HEK293 cells stably expressing the Ca_v_1.2 subunits α_1C_, β_2b_, and α_2_δ_1_ under a tetracycline-inducible promoter (HEK293-Ca_v_1.2) were purchased from B’SYS (Switzerland). Cells were cultured at 37 °C at 5% CO_2_, in Dulbecco’s Modified Eagle Medium/F12 GlutaMAX (Gibco) supplemented with 10% fetal bovine serum, 1× penicillin-streptomycin, 100 μg/ml Hygromycin B, 15 μg/ml Blasticidin, 0.4 μg/ml Puromycin, and 100 μg/ml Zeocin. Cells at approximately 40% confluency were transfected with 1 μg of pHIV- CaM-EGFP variants using Lipofectamine 2000 (Invitrogen) according to the manufacturer’s guidelines. To induce expression of Ca_v_1.2, 2.5 μg/ml tetracycline was applied to cells 24 h prior to experimental use.

#### Electrophysiology

The conventional whole-cell voltage-clamp configuration was used to obtain Ca^2+^ current recordings. Currents were recorded using an Axopatch 200B amplifier (Molecular Devices), filtered at 2 kHz, and sampled at 10 kHz using a Digidata 1320A interface (Molecular Devices). All recordings were taken at room temperature. Internal pipette solution consisted of 140 mM CsMeSO_4_, 0.5 mM EGTA, 10 mM Hepes, 1 mM MgCl_2_, 1 mM Na-ATP, pH adjusted to 7.2 with CsOH. External solutions consisted of 140 mM NaCl, 5 mM CsCl, 0.33 mM NaH_2_PO_4_, 5 mM glucose, 10 mM Hepes, 1 mM MgCl_2_, and 2 mM of either CaCl_2_ or BaCl_2_ for the measurement of Ca^2+^ and Ba^2+^ currents, respectively. pH was adjusted to 7.4 with CsOH. Patch pipettes were pulled from borosilicate glass (outer diameter 1.5 mm, inner diameter 1.17 mm; Harvard Apparatus) and fire-polished to give a resistance of 3 to 5 MΩ once filled with pipette solution. HEK293-Ca_v_1.2 cells transiently transfected with CaM were identified by EGFP fluorescence using a Nikon Eclipse TE200 inverted microscope with epifluorescence attachment. The Ca^2+^ current activation protocol consisted of 300 ms voltage steps ranging from −40 to +60 mV from a holding potential of −60 mV. Steady state inactivation was measured with a protocol comprising a 1 s conditioning prepulse to between −60 mV and +40 mV, from a holding potential of -60 mV, followed by a 300 ms test pulse to +10 mV. Half maximal activation and inactivation (V_50_) were calculated by fitting normalized peak conductance using the Boltzmann equation. For the determination of CDI, the residual current at the end of the 300 ms pulse from the activation protocol (r300) was calculated in extracellular solutions containing either Ca^2+^ (r300_Ca_) or Ba^2+^ (r300_Ba_). The proportion of current inactivation due to CDI (f300) was calculated using the following equation:

$$f300=\frac{r300_{Ba}-r300_{Ca}}{r300_{Ba}}$$

### HEK293T cell culture, transfection, and confocal imaging

HEK293T cells (American Type Culture Collection) were cultured at 37 °C/5% CO_2_, in Dulbecco’s Modified Eagle Medium GlutaMAX (Gibco) supplemented with 10% fetal bovine serum, 1× penicillin-streptomycin (Gibco), and 1× nonessential amino acids (Gibco). For Ca^2+^ imaging experiments, cells were seeded onto 35 mm poly-lysine–treated glass-bottomed dishes (MatTek Corporation). Effectene reagent (Qiagen) was used to cotransfect eGFP-hRyR2 (pcDNA3) and CaM variant (dTomato) plasmids in a 1:2 M ratio, according to the manufacturer's instructions. Posttransfection (48 h), cells were loaded with 10 μM Calbryte 520-AM (AAT Bioquest) for ∼1 h. Then, cells were covered with Krebs Ringer Hepes buffer (25 mM Hepes, 4.8 mM KCl, 120 mM NaCl, 5.5 mM glucose, 1.2 mM KH_2_PO_4_, 1.2 mM MgSO_4_, 1.3 mM CaCl_2_, pH 7.4) and imaging was carried out at on a 3i Marianas spinning-disk. The confocal microscope was equipped with a Zeiss AxioObserver Z1, a 20 × /0.8 Plan-Apochromat air objective, and a 3i Laserstack as an excitation light source (488 nm, for Calbryte/eGFP; 561 nm, for dTomato). Emitted light was collected through single bandpass filters (CSU–X filter wheel, Yokogawa) onto a digital complementary metal-oxide semiconductor camera (Orca-Fusion, Hamamatsu).

Data were acquired at 1024 × 1024 pixel resolution at a rate of ∼5 frames/sec, using SlideBook v.6 software. Spontaneous Ca^2+^ oscillations were recorded from cells coexpressing hRyR2 (eGFP) and CaM variants (dTomato). Calbryte 520 fluorescence signals were measured from regions of interest outlining individual cells using Fiji, and kinetic parameters of Ca^2+^ release were quantified using SignalFind (Dr Antony McCabe, Computational Biology Facility, University of Liverpool).

### Equilibrium Ca^2+^ titrations

Intrinsic tyrosine fluorescence was used to monitor Ca^2+^ binding to the C-lobe of CaM. Proteins (6 μM) were titrated with increasing free [Ca^2+^] in 50 mM Hepes, 100 mM KCl, 1 mM MgCl_2_, 0.5 mM EGTA, 0.5 mM NTA, pH 7.4. Fluorescence emission spectra were recorded at λ_exc_ 277 nm and λ_em_ 300 to 320 nm (peak at 307 nm) on a JASCO FP-6300 spectrofluorometer. Free [Ca^2+^] were calculated using Maxchelator Web Maxc standard (61) to achieve concentrations ranging from 17 nM to 11 mM across 24 titration points.

Fluorescence emission at each Ca^2+^ titration point was normalized to the maximum change in fluorescence for each sample. Data were analyzed on GraphPad Prism and affinity (*K*_d_) was obtained using specific binding with Hill slope nonlinear fitting.

### Secondary structure content

Far-UV CD spectra (180–260 nm) were recorded at 20 °C in a 0.1 cm path length quartz cell using a JASCO J-1100 spectrometer equipped with a JASCO MCB-100 mini circulation bath for temperature control. Purified proteins (10 μM) were measured in 2 mM Hepes (pH 7.5) supplemented with either 1 mM EGTA or 1 mM CaCl_2_ for apo- or Ca^2+^-bound CaM, respectively.

For each sample, three scans were averaged (scan rate 100 nm.min^-1^) and buffer baseline subtracted prior to analysis. Data were normalized to mean residue ellipticity and the secondary structure content was estimated using the CDSSTR prediction algorithm (DichroWeb online server, reference set 7) (110, 111).

### Temperature sensitivity and limited proteolysis

#### Thermal stability

Sensitivity of apo-CaM (Ca^2+^-free) to temperature was assessed by decrease in α-helical content measured by CD. Ellipticity at 222 nm for CaM variants was recorded in a 0.1 cm path length quartz cell using a JASCO J-1100 spectrometer equipped with a JASCO MCB-100 mini circulation bath for temperature control. Purified proteins (10 μM) were measured in 2 mM Hepes (pH 7.5) supplemented with 1 mM EGTA. Temperature ranged from 20 °C to 80 °C in 2 °C increments, with a ramp increase rate of 1 °C/min and a 180s equilibration period between recordings. Data were normalized and fitted to the Boltzmann sigmoid equation (GraphPad Prism) to derive the melting temperature of CaM (Tm).

#### Proteolytic stability

Sensitivity of CaM to enzymatic degradation by trypsin or AspN (New England Biolabs) was assessed by SDS-PAGE and densitometry analysis. Purified CaM (5 μM) were incubated with proteases for 30 min at 37 °C before rapid termination of the reaction by addition of SDS-containing sample buffer and heating to 95 °C for 10 min. Trypsin digestions were performed in 25 mM Hepes, 100 mM NaCl, pH 7.5 with protease concentration 0 to 10 ng/μl in apo conditions (10 mM EGTA) and 0 to 30 ng/μl in Ca^2+^-bound conditions (5 mM CaCl_2_). AspN digestions were performed in 50 mM Tris–HCl, 2.5 mM ZnSO_4_, pH 8 with protease concentration 0 to 5 ng/μl in apo conditions (10 mM EGTA) and 0 to 300 ng/μl in Ca^2+^-bound conditions (5 mM CaCl_2_).

Proteins were separated by NuPAGE 4 to 12% Bis-Tris (Life Technologies) and stained with InstantBlue (Thermo Fisher Scientific). Images were obtained on a ChemiDoc XRS+ transilluminator (Bio-Rad) and the amount of intact CaM was quantified by densitometry using Fiji software.

### Data analysis and statistics

Experiments were performed at least in triplicates and analyzed using GraphPad Prism. Statistical significance levels were obtained using a two-tailed unpaired Student's *t* test, one-way ANOVA, or two-way ANOVA, as described in the appropriate figure legends. *p*-values are represented by stars with ∗*p* < 0.05, ∗∗*p* < 0.01, ∗∗∗*p* < 0.001, and ∗∗∗∗*p* < 0.0001. Structure representations were created using UCSF Chimera software and figures were generated using CorelDRAW 2021. Statistical parameters for all ANOVA tests are presented in Tables S3–S12.

## Data availability

All data have been included within the article and supplementary information. Raw data files are to be shared upon request.

## Supporting information

This article contains supporting information.

## Conflict of interest

The authors declare no competing or financial interests.
