# Supplementary material for: Calmodulin variant E140G associated with long QT syndrome impairs CaMKIIδ autophosphorylation and L-type calcium channel inactivation
Source: J Biol Chem. 2022 Dec 8;299(1):102777. doi: 10.1016/j.jbc.2022.102777 (PMC9830374; doi:10.1016/j.jbc.2022.102777)
Supplement: Supplemental information [file mmc1.docx]

**­­TITLE**

Calmodulin variant E140G associated with long QT syndrome impairs CaMKIIδ auto-phosphorylation and L-type calcium channel (Ca_v_1.2) inactivation

**AUTHORS**

Ohm Prakash^1‡^, Nitika Gupta^2‡^, Amy Milburn^1^, Liam McCormick^1^, Vishvangi Deugi^1^, Pauline Fisch^1^, Jacob Wyles^1^, N Lowri Thomas^3^, Svetlana Antonyuk^4^, Caroline Dart^2^, Nordine Helassa^1*^

^‡^ These authors contributed equally to this work.

^*^ For correspondence: Nordine Helassa, [nhelassa@liverpool.ac.uk](mailto:nhelassa@liverpool.ac.uk).

^1^ Liverpool Centre for Cardiovascular Science, Department of Cardiovascular and Metabolic Medicine, Institute of Life Course and Medical Sciences, Faculty of Health and Life Sciences, University of Liverpool, Liverpool L69 3BX, UK.

^2^ Department of Molecular Physiology and Cell Signalling, Institute of Systems, Molecular and Integrative Biology, Faculty of Health and Life Sciences, University of Liverpool, Liverpool L69 3BX, UK.

^3^ School of Pharmacy & Pharmaceutical Sciences, Cardiff University, Cardiff, Redwood Building, CF10 3NB, UK.

^4^ Molecular Biophysics Group, Institute of Systems, Molecular and Integrative Biology, Faculty of Health and Life Sciences, University of Liverpool, Liverpool L69 7ZB, UK.

**SHORT TITLE**

Calmodulin E140G disrupts CaMKIIδ and Ca_v_1.2 activity

**CLASSIFICATIONS**

Ca^2+^/calmodulin‐dependent protein kinase II (CaMKII)

Calcium‐binding protein

Calcium channel

Calmodulin (CaM)

Cardiovascular disease

**KEYWORDS**

Calmodulin, Ca^2+^/calmodulin‐dependent protein kinase II, CaMKIIδ, cardiac arrhythmia, long QT syndrome, LQTS, L-type voltage-gated Ca^2+^ channel, Ca_v_1.2

**FIGURES**


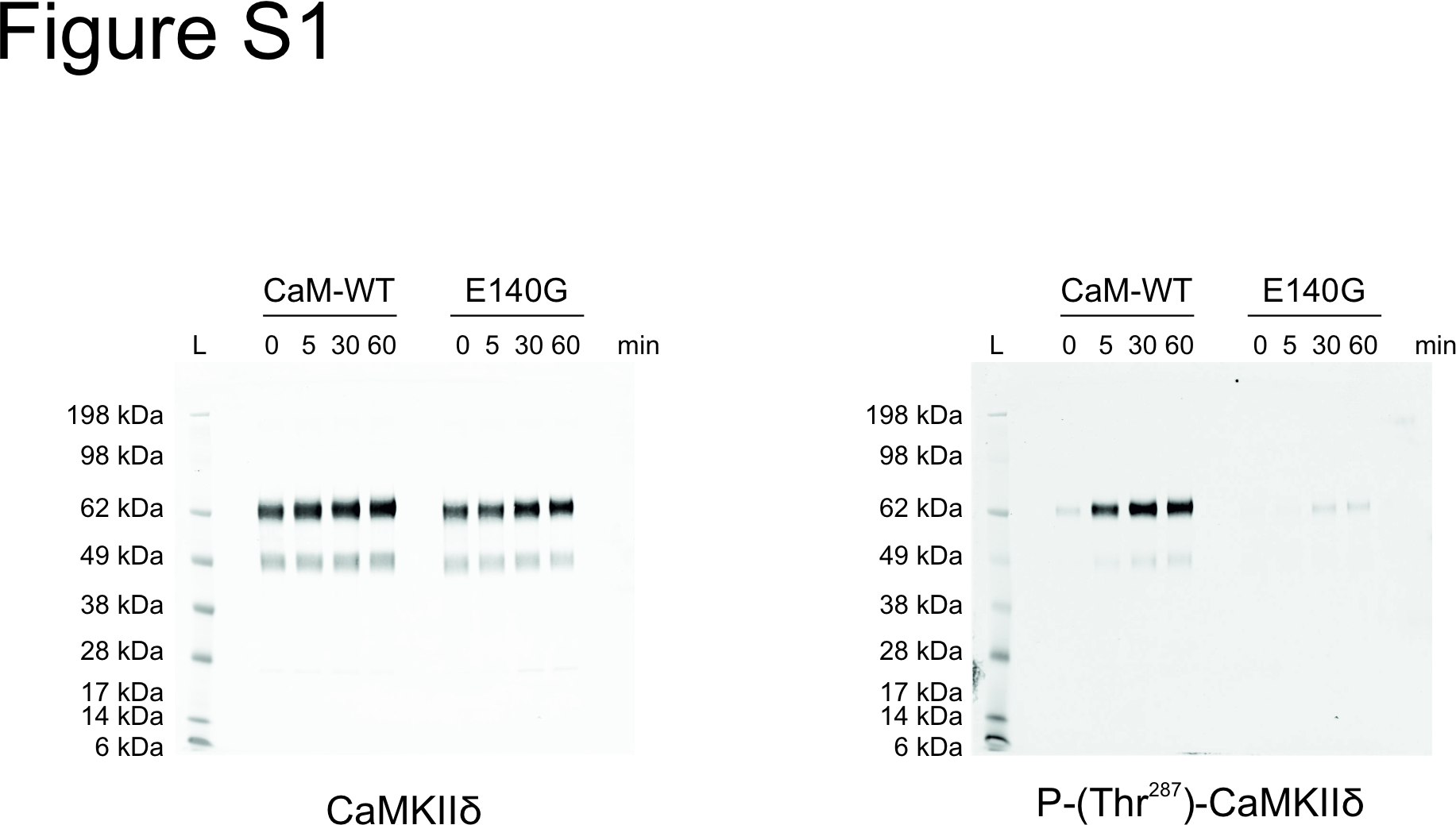


**Figure S1. Full representative western blots for CaM-WT and CaM-E140G samples presented in Figure 1.**

CaM protein (1 µM) and GST-CaMKIIδ (300 nM) were incubated in 50 mM K^+^-HEPES, pH 7.5, 100 mM KCl, 2 mM MgCl_2_, 5 mM DTT, 100 µM CaCl_2_, 300 µM ATP at room temperature. Proteins were separated by SDS-PAGE and analysed by western-blot. Primary antibodies were mouse anti-GST (for CaMKIIδ) and rabbit anti-phospho T287 (for (for phospho-CaMKIIδ). Secondary antibodies were IRDye® 680RD donkey anti-mouse IgG and IRDye® 800CW donkey anti-rabbit IgG. The bands were visualized using an Odyssey CLx infrared imaging system. Densitometry analysis is presented in Figure 1.

**
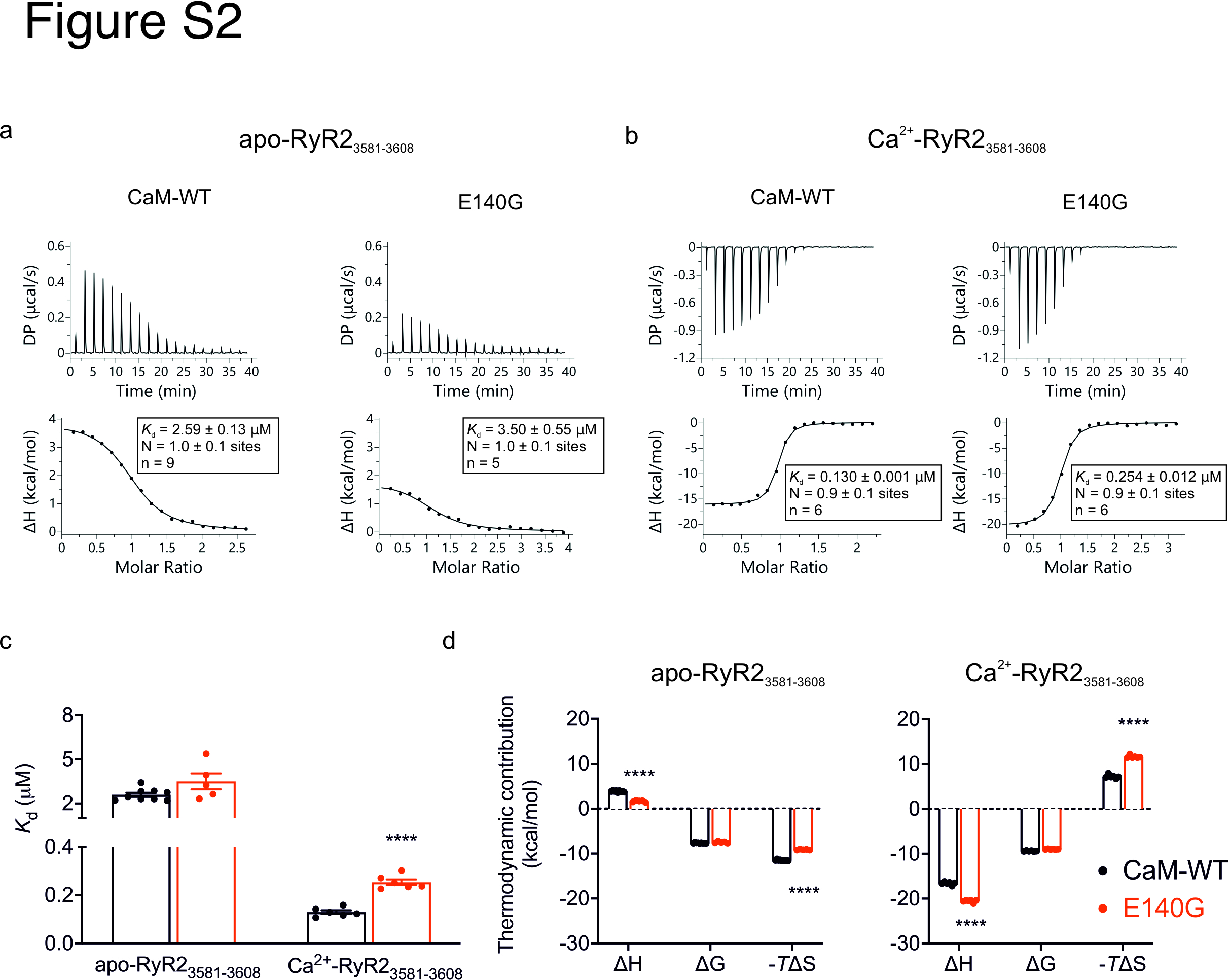
**

**Figure S2. LQTS-associated E140G mutation alters interaction of Ca^2+^-CaM with RyR2 peptide.** (a,b) Representative ITC titration curves (upper panels) and binding isotherms (lower panels) for CaM interaction with RyR2 (a) in the absence and (b) presence of Ca^2+^. DP, differential power. (c) Affinity of the binding of apo-CaM and Ca^2+^/CaM with RyR2 obtained by fitting to a one-site binding model. Differences between groups were determined using a two-tailed unpaired Student *t*-test. (d) Thermodynamic profile of binding between apo-CaM and RyR2 (left panel) and Ca^2+^/CaM and RyR2 (right panel). Data were processed using the MicroCal PEAQ-ITC software. *K*_d_, binding affinity; N, stoichiometry; n, number of experimental replicates. The sum of the change in enthalpy (ΔH) and the change in entropy (ΔS) multiplied by the absolute temperature (T) gives the change in free energy (ΔG). Experiments were performed in the presence of 5 mM EGTA or 5 mM CaCl_2_ at 25 °C. Data are mean±s.e.m. Differences between groups were determined using a two-way ANOVA with Sidak’s multiple comparisons test. P-values are represented by stars with ****P<0.0001. The ANOVA parameters are shown in Table S12.

**
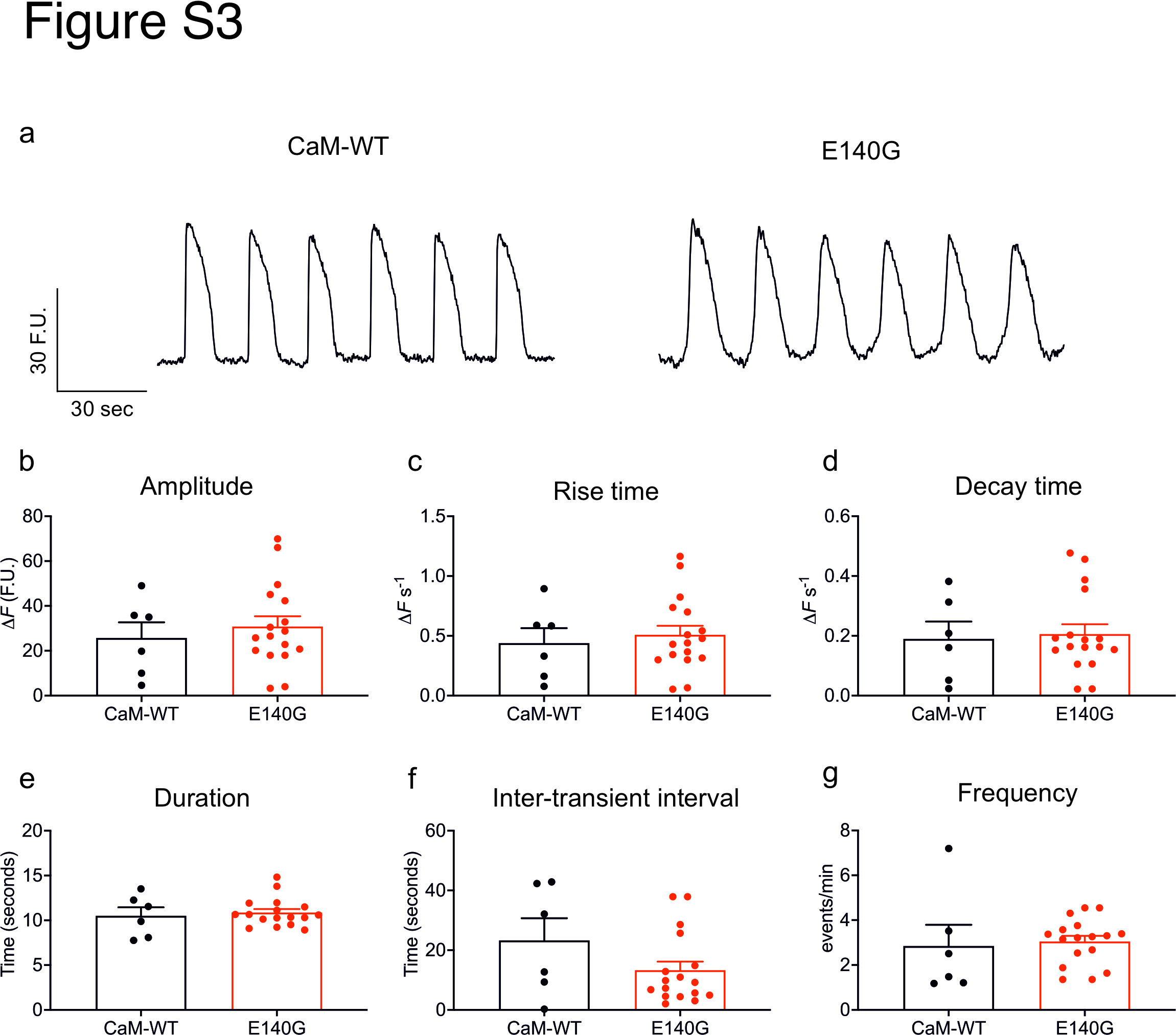
**

**Figure S3. LQTS-associated variant CaM-E140G does not affect RyR2-mediated spontaneous Ca^2+^ transients in cells.**

HEK293T cells transfected with hRyR2 and CaM variants were loaded with the Ca^2+^ dye Calbryte 520 AM to monitor intracellular Ca^2+^ concentration changes. Live cells were imaged on a 3i Marianas spinning-disk confocal microscope. (a) Representative fluorescence signals in HEK293T cells co-expressing hRyR2 and CaM-WT or CaM-E140G. (b-g) Determination of the Ca^2+^ transients kinetic parameters using Fiji and SignalFind softwares. Data are expressed as mean±s.e.m. The numbers of experimental replicates (*N*=dishes, *n*=fields of view) were as follows: *N*=3, *n*=6 for CaM-WT; and *N*=7, *n*=17 for CaM-E140G. Differences between groups were determined using a two-tailed unpaired Student *t*-test. F.U., fluorescence unit; Δ*F*, difference between the maximum fluorescence and the initial fluorescence.

*Amplitude* is determined as the vertical distance from the baseline to the maximum fluorescence value, for each peak (in fluorescence arbitrary units). *Rise time* is calculated from the gradient between baseline and maximum fluorescence, for each peak (in fluorescence arbitrary units per second). *Decay time* is calculated from the gradient between maximum fluorescence value back to the baseline, for each peak (in fluorescence arbitrary units per second). *Duration* is determined as the horizontal distance between the beginning and the end of the peak, for each peak (in seconds). *Inter-transient interval* is determined as the horizontal distance between the end of one peak and the beginning of the next one, for each peak (in seconds). *Frequency* is calculated as the number of peaks per minute.

**
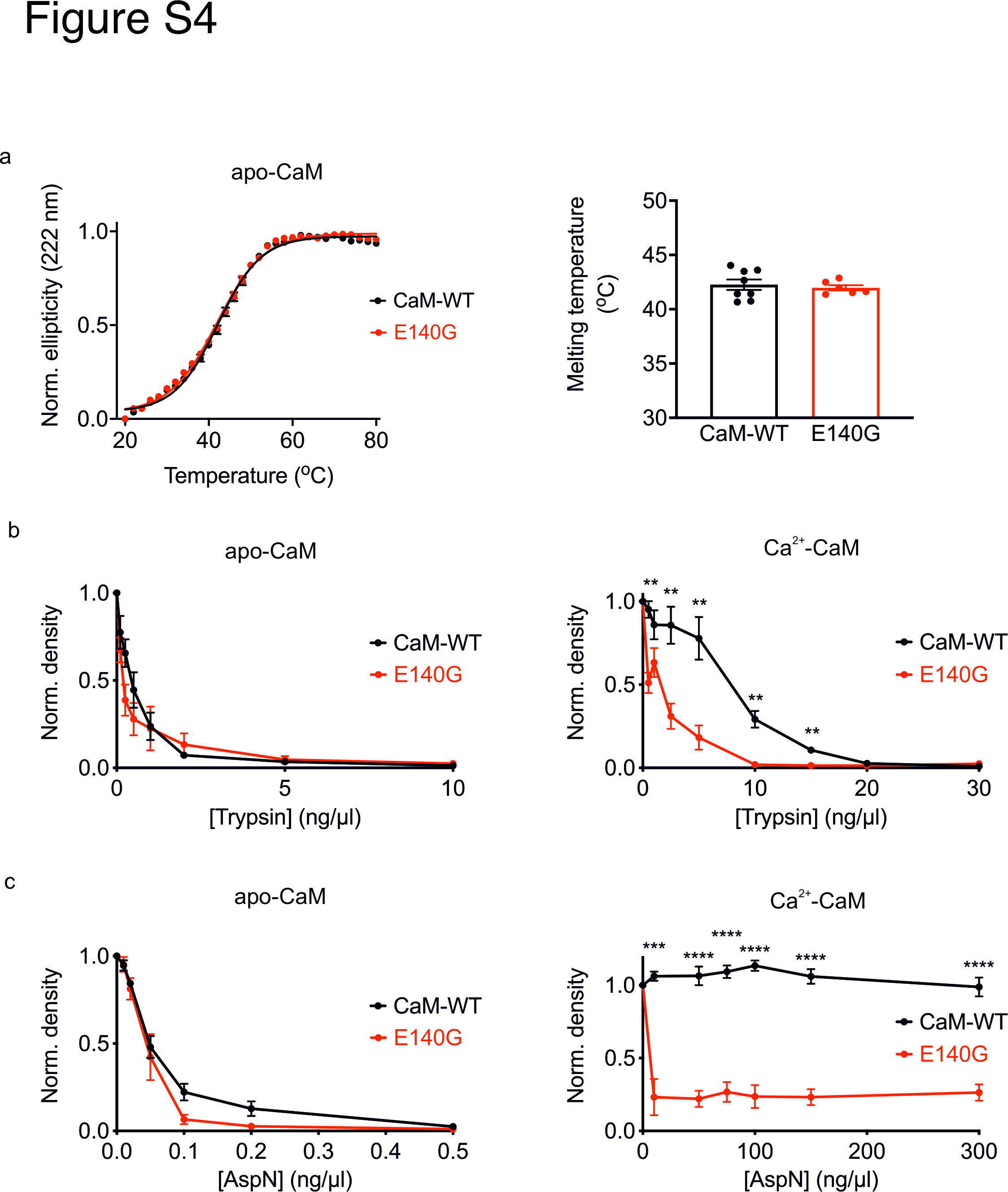
**

**Figure S4. Protease and thermal stability of CaM-WT and CaM-E140G variant.** (a) Thermal unfolding of CaM proteins (10 µM) monitored by circular dichroism recorded at 222 nm from 20 to 80 °C, in the presence of 1 mM EGTA. Data were normalised and expressed as mean±s.e.m. Melting temperature (Tm) was obtained by fitting the traces to the Boltzmann equation. Number of experimental replicates was n=8 for CaM-WT, n=6 for CaM-E140G. (b-c) Limited proteolysis of CaM-WT and CaM-E140G in absence (left) and presence (right) of Ca^2+^. Purified CaM proteins were mixed with increasing concentration of (b) trypsin or (c) AspN for 30 minutes at 37 °C. The fraction of intact CaM was determined by SDS-PAGE and Coomassie staining. Bands were quantified by densitometry analysis using Fiji. Data were normalised and expressed as mean±s.e.m. Number of experimental replicates for trypsin apo-condition: n=7 for CaM-WT, n=4 for CaM-E140G; and for Ca^2+^-saturating condition: n=4 for CaM-WT, n=4 for CaM-E140G. Number of experimental replicates for AspN apo-condition: n=5 for CaM-WT, n=3 for CaM-E140G; and for Ca^2+^-saturating condition: n=5 for CaM-WT, n=4 for CaM-E140G. Differences between groups were determined using a two-tailed unpaired Student *t*-test. P-values are represented by stars with ***P<0.001, ****P<0.0001.

**TABLES**

**Table S1:** Residues from CaMKIIδ peptide (Chain B) and Ca^2+^/CaM-WT (Chain A) forming hydrogen bonds and salt bridges as per QtPISA interfaces prediction.

| **Hydrogen Bonds** | |
| --- | --- |
| **CaMKIIδ Peptide** | **CaM-WT** |
| Arg 4 [NH1] | Glu 127 [O] |
| Arg 4 [NH2] | Met 144 [O] |
| Thr 13 [OG1] | Ala 15 [O] |
| Met 15 [O] | Lys 75 [NZ] |
| **Salt Bridges** | |
| **CaMKIIδ Peptide** | **CaM-WT** |
| Arg 4 [NH1] | Glu 127 [OE1] |
| Arg 5 [NE] | Glu 7 [OE1] |
| Arg 5 [NH2] | Glu 7 [OE2] |
| Lys 6 [NZ] | Glu 114 [OE1] |
| Lys 8 [NZ] | Glu 11 [OE1], [OE2] |
| Arg 19 [NH1] | Glu 84 [OE2] |
| Ser 22 [O] [OXT] | Lys 75 [NZ] |

**Table S2:** Residues from CaMKIIδ peptide (Chain B) and Ca^2+^/CaM-E140G (Chain A) variant forming hydrogen bonds and salt bridges as per QtPISA interfaces prediction.

| **Hydrogen Bonds** | |
| --- | --- |
| **CaMKIIδ Peptide** | **CaM-E140G** |
| Arg 4 [NH1] | Glu 127 [O] |
| Arg 4 [NE] | Ala 147 [O] |
| Lys 8 [NZ] | Ala 147 [O] |
| Thr 13 [OG1] | Ala 15 [O] |
| Thr 18 [OG1] | Glu 87 [OE1] |
| **Salt Bridges** | |
| **CaMKIIδ Peptide** | **CaM-E140G** |
| Arg 4 [NH1] | Glu 127 [OE1] |
| Arg 5 [NE], [NH2] | Glu 14 [OE1] |
| Lys 6 [NZ] | Glu 14 [OE2] |
| Lys 6 [NZ] | Glu 114 [OE1] |
| Lys 8 [NZ] | Glu 11 [OE2] |
| Lys 8 [NZ] | Glu 7 [OE1] |

**Table** **S3:** Report of the statistical parameters for the two-way ANOVA test performed in Figure 2i.

|  | **F** | **P value** |
| --- | --- | --- |
| **Row factor**  Thermodynamic parameters  (CaMKIIδ_294-315_) | 1668 | <0.0001 |
| **Column factor**  Calmodulin variant | 1.883 | =0.1793 |
| **Interaction** | 80.52 | <0.0001 |

| **Šídák's multiple comparisons test** | **Summary** | **P value** |
| --- | --- | --- |
|  |  |  |
| CaM-WT vs. E140G  (CaMKIIδ_294-315_) |  |  |
| ΔH | **** | <0.0001 |
| ΔG | ns | 0.5574 |
| -*T*ΔS | **** | <0.0001 |

**Table** **S4:** Report of the statistical parameters for the two-way ANOVA test performed in Figure 3b.

|  | **F** | **P value** |
| --- | --- | --- |
| **Row factor**  Voltage | 53.90 | <0.0001 |
| **Column factor**  Calmodulin variant | 1.830 | =0.1813 |
| **Interaction** | 1.377 | =0.1338 |

| **Tukey's multiple comparisons test** | **Summary** | **P value** |
| --- | --- | --- |
|  |  |  |
| **Row 1: - 40 mV** |  |  |
| endogenous vs. CaM-WT | ns | 0.5234 |
| endogenous vs. E140G | ns | 0.6442 |
| CaM-WT vs. E140G | ns | 0.0898 |
|  |  |  |
| **Row 2: - 30 mV** |  |  |
| endogenous vs. CaM-WT | ns | 0.8475 |
| endogenous vs. E140G | ns | 0.6242 |
| CaM-WT vs. E140G | ns | 0.0520 |
|  |  |  |
| **Row 3: - 20 mV** |  |  |
| endogenous vs. CaM-WT | ns | 0.7389 |
| endogenous vs. E140G | ns | 0.3159 |
| CaM-WT vs. E140G | ns | 0.3859 |
|  |  |  |
| **Row 4: - 10 mV** |  |  |
| endogenous vs. CaM-WT | ns | 0.7026 |
| endogenous vs. E140G | ns | 0.2054 |
| CaM-WT vs. E140G | ns | 0.3324 |
|  |  |  |
| **Row 5: 0 mV** |  |  |
| endogenous vs. CaM-WT | ns | 0.6418 |
| endogenous vs. E140G | ns | 0.2111 |
| CaM-WT vs. E140G | ns | 0.1710 |
|  |  |  |
| **Row 6: + 10 mV** |  |  |
| endogenous vs. CaM-WT | ns | 0.8623 |
| endogenous vs. E140G | ns | 0.2649 |
| CaM-WT vs. E140G | ns | 0.0972 |
|  |  |  |
| **Row 7: + 20 mV** |  |  |
| endogenous vs. CaM-WT | ns | 0.9900 |
| endogenous vs. E140G | ns | 0.3395 |
| CaM-WT vs. E140G | ns | 0.1091 |
|  |  |  |
| **Row 8: + 30 mV** |  |  |
| endogenous vs. CaM-WT | ns | 0.9995 |
| endogenous vs. E140G | ns | 0.4035 |
| CaM-WT vs. E140G | ns | 0.1999 |
|  |  |  |
| **Row 9: + 40 mV** |  |  |
| endogenous vs. CaM-WT | ns | 0.9854 |
| endogenous vs. E140G | ns | 0.4523 |
| CaM-WT vs. E140G | ns | 0.5197 |
|  |  |  |
| **Row 10: + 50 mV** |  |  |
| endogenous vs. CaM-WT | ns | 0.9235 |
| endogenous vs. E140G | ns | 0.5607 |
| CaM-WT vs. E140G | ns | 0.8886 |
|  |  |  |
| **Row 11: + 60 mV** |  |  |
| endogenous vs. CaM-WT | ns | 0.8137 |
| endogenous vs. E140G | ns | 0.9128 |
| CaM-WT vs. E140G | ns | 0.9112 |

**Table** **S5:** Report of the statistical parameters for the one-way ANOVA test performed in Figure 3c.

|  | **F** | **P value** |
| --- | --- | --- |
| **Column factor**  Calmodulin variant | 0.3475 | =0.7098 |

| **Dunnett’s multiple comparisons test** | **Summary** | **P value** |
| --- | --- | --- |
| CaM-WT vs. endogenous | ns | 0.6368 |
| CaM-WT vs. E140G | ns | 0.9456 |

**Table** **S6:** Report of the statistical parameters for the one-way ANOVA test performed in Figure 3d.

|  | **F** | **P value** |
| --- | --- | --- |
| **Column factor**  Calmodulin variant | 0.3475 | =0.7098 |

| **Dunnett’s multiple comparisons test** | **Summary** | **P value** |
| --- | --- | --- |
| CaM-WT vs. endogenous | ns | 0.5798 |
| CaM-WT vs. E140G | ns | 0.1968 |

**Table** **S7:** Report of the statistical parameters for the one-way ANOVA tests performed in Figure 4c.

*In Ca^2+^*

|  | **F** | **P value** |
| --- | --- | --- |
| **Column factor**  Calmodulin variant  (Ca^2+^) | 67.34 | <0.0001 |

| **Dunnett’s multiple comparisons test** | **Summary** | **P value** |
| --- | --- | --- |
| CaM-WT vs. endogenous | ns | 0.6517 |
| CaM-WT vs. E140G | **** | <0.0001 |

*In Ba^2+^*

|  | **F** | **P value** |
| --- | --- | --- |
| **Column factor**  Calmodulin variant  (Ba^2+^) | 0.2290 | =0.7985 |

| **Dunnett’s multiple comparisons test** | **Summary** | **P value** |
| --- | --- | --- |
| CaM-WT vs. endogenous | ns | 0.7365 |
| CaM-WT vs. E140G | ns | 0.7975 |

**Table** **S8:** Report of the statistical parameters for the one-way ANOVA test performed in Figure 4d.

|  | **F** | **P value** |
| --- | --- | --- |
| **Column factor**  Calmodulin variant | 49.18 | <0.0001 |

| **Dunnett’s multiple comparisons test** | **Summary** | **P value** |
| --- | --- | --- |
| CaM-WT vs. endogenous | ns | 0.7909 |
| CaM-WT vs. E140G | **** | <0.0001 |

**Table** **S9:** Report of the statistical parameters for the two-way ANOVA tests performed in Figure 5d.

*For Ca_v_1.2-NSCaTE_51-67_*

|  | **F** | **P value** |
| --- | --- | --- |
| **Row factor**  Thermodynamic parameters (Ca_v_1.2-NSCaTE_51-67_) | 3318 | <0.0001 |
| **Column factor**  Calmodulin variant | 14.30 | =0.0009 |
| **Interaction** | 649.4 | <0.0001 |

| **Šídák's multiple comparisons test** | **Summary** | **P value** |
| --- | --- | --- |
|  |  |  |
| CaM-WT vs. E140G  (Ca_v_1.2-NSCaTE_51-67_) |  |  |
| ΔH | **** | <0.0001 |
| ΔG | * | 0.0101 |
| -*T*ΔS | **** | <0.0001 |

*For Ca_v_1.2-IQ_1665-1685_*

|  | **F** | **P value** |
| --- | --- | --- |
| **Row factor**  Thermodynamic parameters (Ca_v_1.2-IQ_1665-1685_) | 854.4 | <0.0001 |
| **Column factor**  Calmodulin variant | 1.222 | =0.2748 |
| **Interaction** | 0.4593 | =0.6346 |

| **Šídák's multiple comparisons test** | **Summary** | **P value** |
| --- | --- | --- |
|  |  |  |
| CaM-WT vs. E140G  (Ca_v_1.2-IQ_1665-1685_) |  |  |
| ΔH | ns | 0.6355 |
| ΔG | ns | 0.7047 |
| -*T*ΔS | ns | 0.9986 |

**Table** **S10:** Report of the statistical parameters for the two-way ANOVA test performed in Figure 5e.

*For CaM–Ca_v_1.2-IQ_1665-1685_:Ca_v_1.2-NSCaTE_51-67_*

|  | **F** | **P value** |
| --- | --- | --- |
| **Row factor**  Thermodynamic parameters  (CaM–Ca_v_1.2-IQ_1665-1685_:Ca_v_1.2-NSCaTE_51-67_) | 135.5 | <0.0001 |
| **Column factor**  Calmodulin variant | 1.312 | =0.2671 |
| **Interaction** | 27.11 | <0.0001 |

| **Šídák's multiple comparisons test** | **Summary** | **P value** |
| --- | --- | --- |
|  |  |  |
| CaM-WT vs. E140G  (CaM–Ca_v_1.2-IQ_1665-1685_:Ca_v_1.2-NSCaTE_51-67_) |  |  |
| ΔH | *** | 0.0005 |
| ΔG | ns | 0.7075 |
| -*T*ΔS | **** | <0.0001 |

**Table** **S11:** Report of the statistical parameters for the two-way ANOVA tests performed in Figure 6c.

*For apo-CaM*

|  | **F** | **P value** |
| --- | --- | --- |
| **Row factor**  Secondary structure type | 573.2 | <0.0001 |
| **Column factor**  Calmodulin variant  (apo-CaM) | 0.04645 | =0.8306 |
| **Interaction** | 0.6361 | =0.5967 |

| **Šídák's multiple comparisons test** | **Summary** | **P value** |
| --- | --- | --- |
|  |  |  |
| CaM-WT vs. E140G  (apo) |  |  |
| α-helix | ns | 0.5989 |
| β-sheet | ns | 0.9998 |
| Turns | ns | 0.9963 |
| Unordered | ns | 0.9907 |

*For Ca^2+^-CaM*

|  | **F** | **P value** |
| --- | --- | --- |
| **Row factor**  Secondary structure type | 1575 | <0.0001 |
| **Column factor**  Calmodulin variant  (Ca^2+^-CaM) | 0.0021 | =0.9636 |
| **Interaction** | 23.68 | <0.0001 |

| **Šídák's multiple comparisons test** | **Summary** | **P value** |
| --- | --- | --- |
|  |  |  |
| CaM-WT vs. E140G  (Ca^2+^) |  |  |
| α-helix | **** | <0.0001 |
| β-sheet | * | 0.0484 |
| Turns | ns | 0.5820 |
| Unordered | ** | 0.0085 |

**Table** **S12:** Report of the statistical parameters for the two-way ANOVA tests performed in Supplementary Figure S2d.

*For apo-RyR2_3581-3608_*

|  | **F** | **P value** |
| --- | --- | --- |
| **Row factor**  Thermodynamic parameters  (apo-RyR2_3581-3608_) | 31279 | <0.0001 |
| **Column factor**  Calmodulin variant | 5.727 | =0.0220 |
| **Interaction** | 867.3 | <0.0001 |

| **Šídák's multiple comparisons test** | **Summary** | **P value** |
| --- | --- | --- |
|  |  |  |
| CaM-WT vs. E140G  (apo-RyR2_3581-3608_) |  |  |
| ΔH | **** | <0.0001 |
| ΔG | ns | 0.1426 |
| -*T*ΔS | **** | <0.0001 |

*For Ca^2+^-RyR2_3581-3608_*

|  | **F** | **P value** |
| --- | --- | --- |
| **Row factor**  Thermodynamic parameters  (Ca^2+^-RyR2_3581-3608_) | 31272 | <0.0001 |
| **Column factor**  Calmodulin variant | 8.584 | =0.0064 |
| **Interaction** | 679.1 | <0.0001 |

| **Šídák's multiple comparisons test** | **Summary** | **P value** |
| --- | --- | --- |
|  |  |  |
| CaM-WT vs. E140G  (Ca^2+^-RyR2_3581-3608_) |  |  |
| ΔH | **** | <0.0001 |
| ΔG | ns | 0.0565 |
| -*T*ΔS | **** | <0.0001 |
